# Multiscale modeling of collective cell migration elucidates the mechanism underlying tumor–stromal interactions in different spatiotemporal scales

**DOI:** 10.1038/s41598-022-20634-5

**Published:** 2022-09-28

**Authors:** Zarifeh Heidary, Shaghayegh Haghjooy Javanmard, Iman Izadi, Nasrin Zare, Jafar Ghaisari

**Affiliations:** 1 Department of Electrical and Computer Engineering, Isfahan University of Technology, Isfahan, 84156-83111 Iran; 2 Department of Physiology, Applied Physiology Research Center, Isfahan Cardiovascular Research Institute, Isfahan University of Medical Sciences, Isfahan, 81746-73461 Iran; 3 School of Medicine, Najafabad Branch, Islamic Azad University, Isfahan, Iran

**Keywords:** Computational biology and bioinformatics, Systems biology

## Abstract

Metastasis is the pathogenic spread of cancer cells from a primary tumor to a secondary site which happens at the late stages of cancer. It is caused by a variety of biological, chemical, and physical processes, such as molecular interactions, intercellular communications, and tissue-level activities. Complex interactions of cancer cells with their microenvironment components such as cancer associated fibroblasts (CAFs) and extracellular matrix (ECM) cause them to adopt an invasive phenotype that promotes tumor growth and migration. This paper presents a multiscale model for integrating a wide range of time and space interactions at the molecular, cellular, and tissue levels in a three-dimensional domain. The modeling procedure starts with presenting nonlinear dynamics of cancer cells and CAFs using ordinary differential equations based on TGFβ, CXCL12, and LIF signaling pathways. Unknown kinetic parameters in these models are estimated using hybrid unscented Kalman filter and the models are validated using experimental data. Then, the principal role of CAFs on metastasis is revealed by spatial–temporal modeling of circulating signals throughout the TME. At this stage, the model has evolved into a coupled ODE–PDE system that is capable of determining cancer cells’ status in one of the quiescent, proliferating or migratory conditions due to certain metastasis factors and ECM characteristics. At the tissue level, we consider a force-based framework to model the cancer cell proliferation and migration as the final step towards cancer cell metastasis. The ability of the multiscale model to depict cancer cells’ behavior in different levels of modeling is confirmed by comparing its outputs with the results of RT PCR and wound scratch assay techniques. Performance evaluation of the model indicates that the proposed multiscale model can pave the way for improving the efficiency of therapeutic methods in metastasis prevention.

## Introduction

Cancer cell metastasis is a key step of cancer progression that accounts for the majority of patient deaths^1,2^. Despite the inherent complexity of metastasis stages, recent studies show that not only intracellular reactions in cancer cells, but also mutual interactions between cancer cells and tumor microenvironment (TME) orchestrate the events during a metastasis cascade^3,4^. In other words, studying the behavior and characteristics of cancer cells without considering their interactions with TME components such as extracellular matrix (ECM), blood vessels, immune cells, adipose cells, and fibroblasts does not provide comprehensive information about the causes and circumstances of tumor cells movement^5,6^.

Cancer associated fibroblasts (CAFs) as one of the abundant factors in TME have a major impact on tumor behavior and they are known as principal participants in tumor growth and invasion^7,8^. They directly contribute to cancer cell proliferation, tumor growth, and invasion through stimulation of various growth factors and chemokines such as transforming growth factor β (TGFβ), vascular endothelial growth factor (VEGF), leukemia inhibitory factor (LIF), and C-X-C motif chemokine 12 (CXCL12) in context-dependent manner^8–10^. These factors are responsible for initiation of signaling pathways and consequent biochemical reactions in both cancer cells and CAFs -as a principle provocative components of tumor metastasis- which lead to tumor progression and metastasis. To address the effects of CAFs on tumor metastasis, it is necessary to investigate the communications between CAFs and cancer cells, as well as their intercellular interactions which result in tumor invasion.

Cancer metastasis is a complex multiscale process in which reactions occur in different time and space scales^11^. Furthermore, the number of these interactions are relatively high and the related signaling pathways usually have crosstalk with each other. Therefore, an advanced tool is required not only for detailed study of these interactions, but also for efficient analysis of the involved mechanisms in different stages of metastasis. This is where systems biology comes into play and provides useful techniques for investigating the complex biological behaviors such as mathematical modeling approaches. Using these methods, we obtain the opportunity to understand the behavioral kinetics inside the cells as well as communications between different cell types in TME. Moreover, mathematical modeling tools enable us to explain how interactions through three different scales including molecular, cellular, and population lead to tumor migration.

Over the last decades, various mathematical modeling approaches have been applied for revealing tumor initiation and growth mechanisms^12–17^. However, the number of studies that model metastasis based on communication of tumor and its milieu, are relatively low. In^18^, a mathematical model has been presented for describing cancer cell intravasation. Using a multiscale approach and a hypothetical framework for biological shape of blood vessel, the model represents the effect of cadherin protein pathways in cancer cell migration.

Also, the role of ECM remodeling in cancer cell invasion by means of degradation enzymes has been investigated in some recent studies^19–21^. Kim et al.^22^ developed a hybrid model that incorporates the interactions of stromal and cancer cells in TME to show how theses communications play role in early development of tumor and its invasion to the stroma. In addition, a number of mathematical models based on agent-based methods have been applied for determining the interactions of platelet cells, stem cells, and tumor cells, as well as the role of immune cells in cancer metastasis^23–26^.

It should be noted that there are a limited number of studies based on mathematical modeling that focus on the effects of CAFs on cancer cells’ behavior. For example, based on the singular value decomposition approach, regulatory rules for interactions of cancer cells and adjacent fibroblasts were modeled in^27^. In addition, a mathematical model for the transformation of fibroblasts into myofibroblasts in TME, as well as the effect of myofibroblasts on tumor cell proliferation via Epidermal growth factor (EGF) release, has been presented^28^. Furthermore, in a recent study we presented an agent-based model of fibroblast switching behavior in tumor suppression and progression in normal and active states^29^. Nonetheless, the mutual conversation between CAFs and tumor cells at microscopic and macroscopic levels that leads to metastasis has never been mathematically investigated.

In the current study, we present a multiscale model of cancer cell metastasis due to their interactions with TME components such as CAFs and ECM. At first, a mathematical modeling framework has been developed to represent the dynamics of cancer cells and CAFs individually based on ordinary differential equations (ODEs). The dynamical models represent biochemical reactions in TGFβ, LIF, and CXCL12 signaling pathways which were retrieved from the literature. As a result, each cell type has its own set of ODEs at the molecular level. Similar to our previous work in^29^, hybrid unscented Kalman filter (HUKF) is used to unravel the unknown parameters as a reliable parameter estimation approach^30^. Then, using partial differential equations (PDEs) for describing the spatial distribution of molecules throughout the TME, we extended the ODE systems to a coupled ODE–PDE system. This coupled system can explain the communications between cancer cells, CAFs and ECM in a three-dimensional space. The proposed model can explain the nonlinear effect of CAFs on the invasive activity of cancer cells at the cellular level.

Moreover, the coupled ODE–PDE system can designate the state of each individual cancer cell as quiescent, proliferative, or migratory. The states of each cell are determined due to the concentration level of some significant factors in signaling pathways such as CXCL12, and SNAIL. Finally, we developed a multiscale model which illustrates the movement of cancer cells in a three dimensions domain using a coupled ODE–PDE system and a biomechanical model at population level. This model represents the movement of cells due to the forces applied to them. Finally, the model is validated in both molecular and population levels by comparing the simulation results with the data extracted from RT PCR and migration assay. The proposed nonlinear multiscale model can not only be utilized in determining the role of CAFs in metastasis, but also might pave the way to finding advanced cancer treatment methods.

## Material and methods

### Multiscale model of cancer metastasis

**Figure 1:**
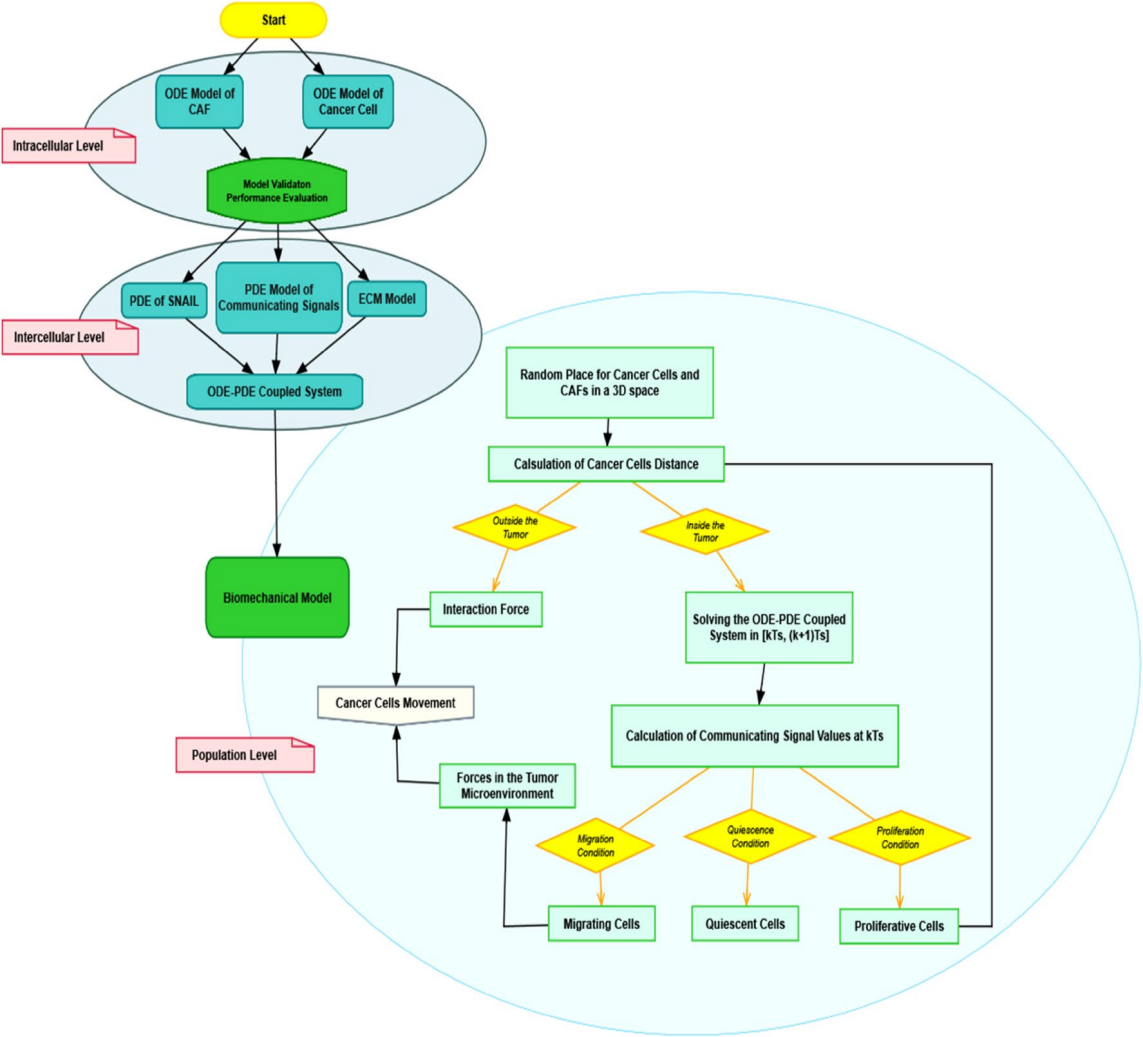
Flowchart of necessary steps in each level of multiscale model of cancer cell metastasis.

The proposed model in this study is comprised of multiple layers each representing interactions at the molecular (within a cell), cellular (between cells), and population levels. The multi-layer model of cancer cell metastasis includes interactions in different components such as cancer cells, CAFs, and their mutual interactions in the TME. At the molecular level, nonlinear ODE models are used to represent the dynamics of TME components which are based on reactions in some signal transduction pathways. Then, the cellular level is basically characterized by the signaling molecules between cancer cells and CAFs. These signals show the impacts of cancer cells on CAF functioning and consequently, the effects of CAFs on cancer cell invasion. In this level, the dynamics show how the behavior of TME components are controlled under the influence of each other. Finally, cancer cell migration is depicted in three dimensions space using a Newtonian dynamic at the population level of the model. In order to model the movement of cancer cells, different layers are connected to each other appropriately. The various levels of modeling technique are given below. Figure 1 depicts the various levels of our multiscale model as well as the actions required at each level to model cancer cell metastasis.

### Dynamical modeling of intracellular interactions within cancer cells and CAFs

At the molecular level, we present two unique nonlinear ODE models for characterizing the dynamics of cancer cell and CAF as two different cell types in TME. Unlike normal cells, some signaling pathways which are responsible for cell growth, proliferation, and motion function abnormally in tumor cells^31^. As a result, we can obtain abundant information from the well-known growth factor expression variations and their linked signaling pathways for better understanding cancer mechanisms. Due to vital roles of TGFβ, LIF and CXCL12 signal transduction pathways in tumor growth, proliferation, motility, and invasion, the ODE models are based on biochemical reactions involved in these signaling pathways^32–35^. They are also the key activators of fibroblasts in TME and abundant within both cancer cells and CAFs^36,37^.

**Figure 2:**
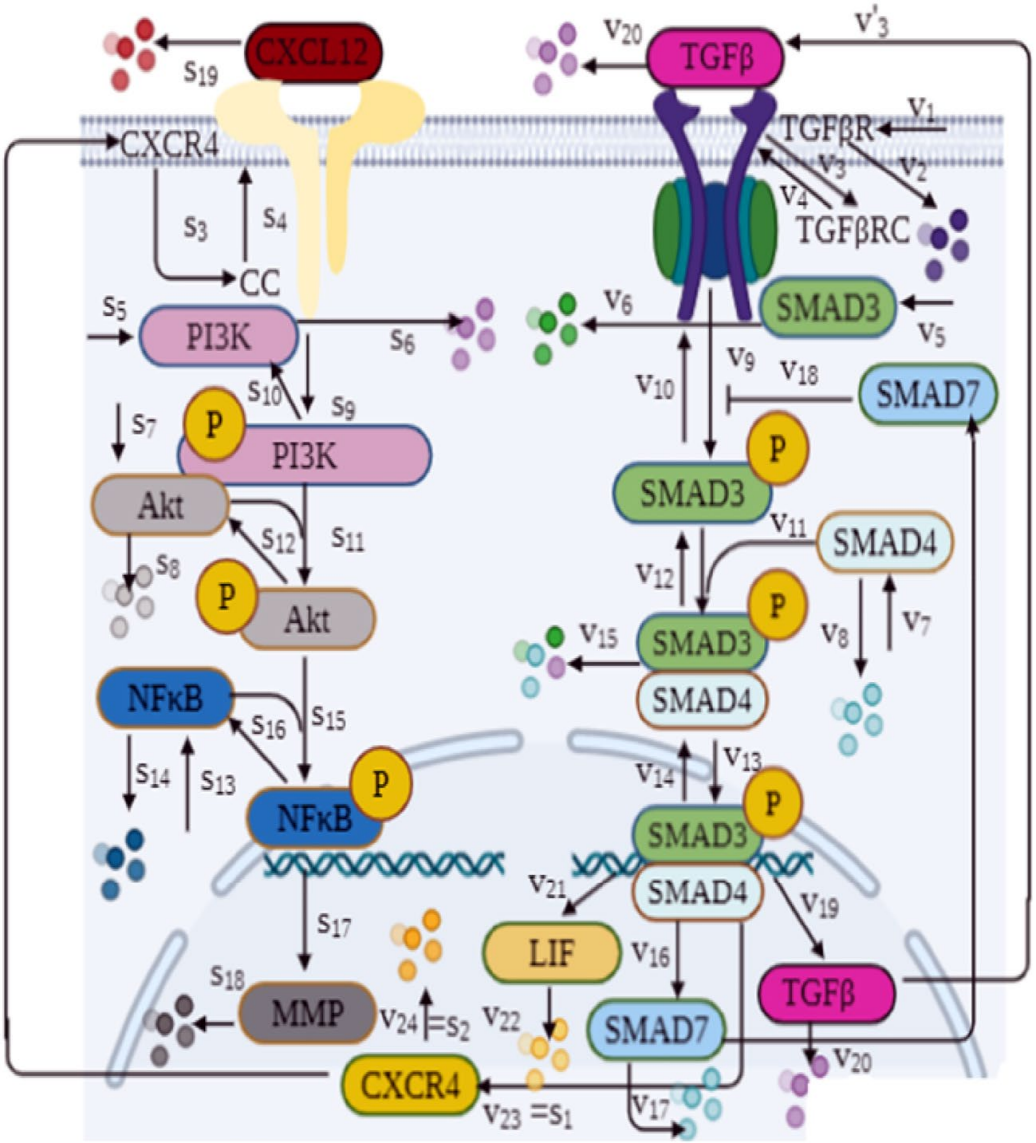
Biochemical reactions involved in dynamical model of TGFβ and CXCL12 pathways as well as their crosstalk within a cancer cell. The name of each reaction is shown on the corresponding arrow. More information about the mathematical representation of reactions are included in Tables 1, S1, and S3.

**Figure 3:**
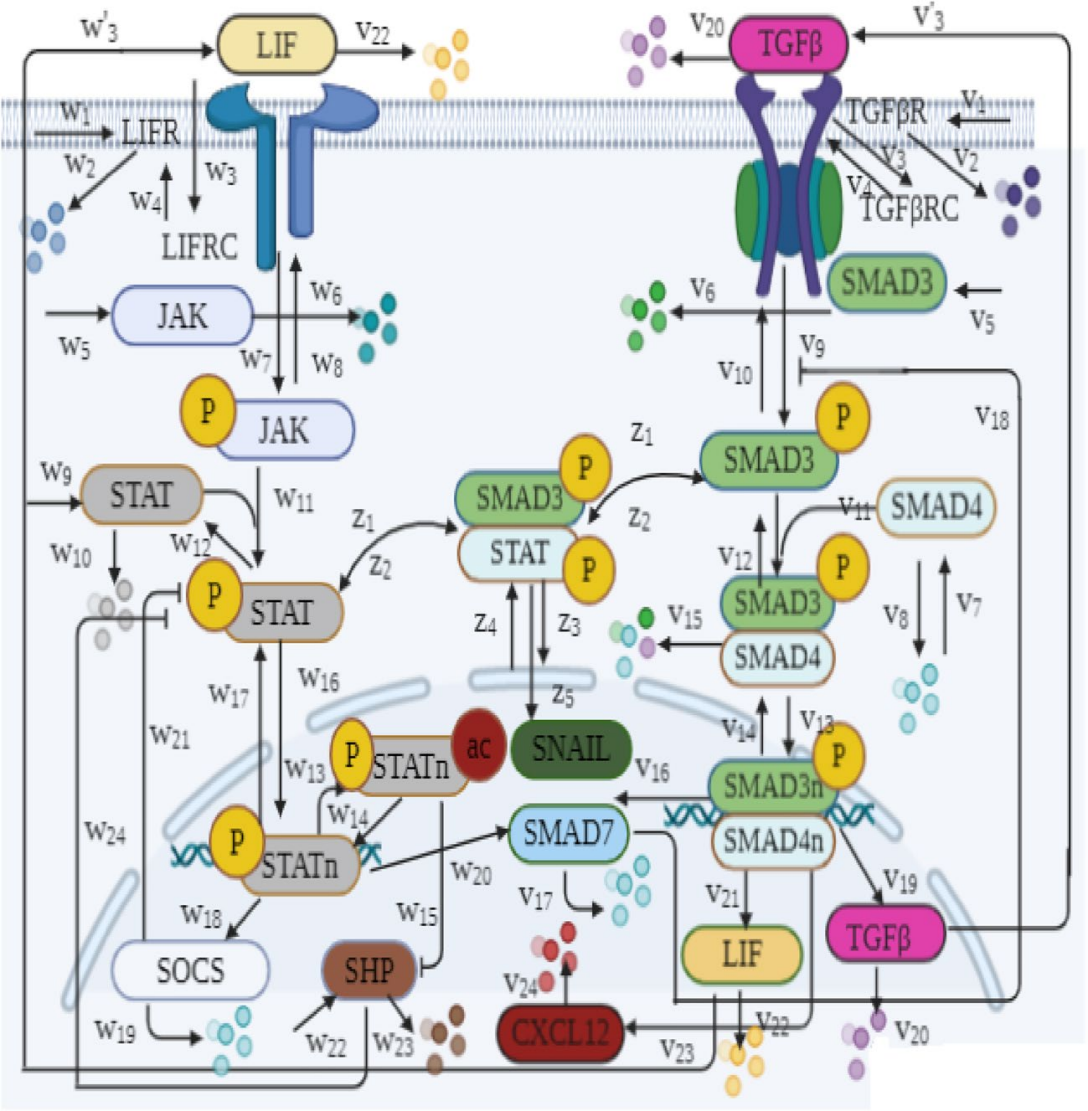
Biochemical reactions involved in dynamic model of TGFβ and LIF pathways as well as their crosstalk within the CAFs. The name of each reaction has been shown on the corresponding arrow. More information about the mathematical representation of reactions are included in Tables 2, S2, and S4.

As demonstrated in Figs. 2 and 3, dynamical models are mathematical representations of biochemical reactions in TGFβ and CXCL12 pathways, as well as TGFβ and LIF signaling cascades with their crosstalk for describing the dynamics of cancer cells and CAFs, respectively. TGFβ, as one of the important factors released in the TME by both CAFs and cancer cells, binds to its receptors (TGFβRs) and consequently initiates a chain of reactions inside the cell. The constitution of active ligand-receptor compounds leads to phosphorylation of SMAD2/3 factors. The phosphorylated SMAD2/3 and SMAD4 then link together to create a heterodimeric complex. This complex imports to the nucleus and acts as a transcription factor for downstream genes such as SMAD7 and Zinc finger protein SNAI1 (SNAIL). SMAD7 inhibits SMAD2/3 phosphorylation upstream in the pathway, resulting in a negative regulatory feedback in TGFβ signaling^38,39^. It should be noted that LIF, C-X-C chemokine receptor type 4 (CXCR4), CXCL12, and TGFβ itself are expressed as the target genes of the pathway^32,33,40,41^. Using this fact, we can conclude that TGFβ interferes in LIF and CXCL12 signaling pathways by increasing the level of these factors, and also it can drive its pathway in both paracrine and autocrine fashions^32,42^. These two points are reflected in our modeling procedure.

CXCL12 signaling pathway begins when this factor reaches its cell surface receptor, CXCR4. The binding of CXCL12 to its receptor prompts various gene expression and regulatory mechanisms through different intermediate proteins inside the cells. Phosphoinositide 3-kinases/ protein kinase B (PI3K/ PKB or Akt) mediator pathway initiate its activity in response to CXCL12 ligand-receptor phosphorylating action. It performs key functions such as controlling cell growth, proliferation, and migration by stimulating transcription factors such as Nuclear factor kappa-light-chain-enhancer of activated B cells (NFκB)^41,43–45^. Then, agitation of NFκB leads to overexpression of matrix metalloproteinases (MMPs) which are the major destructive factors of ECM^46^. ECM degrading or remodeling is a necessity for cancer cell invasion^47,48^. Thus, as it turns out, CXCL12-CXCR4 pathway is selected as a fundamental pathway for dynamical modeling of cancer cells. In our proposed model, CXCL12 pathway is governed by inducing CXCL12 and CXCR4 from CAFs and cancer cells, respectively.

LIF is the other abundant pro-invasive factor in the TME whose overexpression associates with poor clinical results for cancer patients. It displays strong upregulation as a target gene of TGFβ pathway. Moreover, it mediates fibroblasts reprograming into CAFs^36,37^. So, in the presented model, we consider LIF signaling pathway within CAFs in both paracrine and autocrine manners. Janus kinase/ signal transducer and activator of transcription (JAK/STAT) intermediate pathway is driven immediately after binding of LIF to its cell surface receptor. Then, the activated STAT enters nucleus and consequently contributes in transcription of target genes such as suppressor of cytokine signaling (SOCS) and SMAD7^37,49,50^. These two factors both regulate signal transduction by negative feedback creation in LIF and TGFβ pathways. As stated above, SMAD7 inhibits phosphorylation of SMAD2/3 and similarly STAT activation is prevented by SOCS protein^51^. There is another negative regulator for LIF pathway named small heterodimer partner (SHP) proteins which inhibits STAT phosphorylation^10^. Furthermore, if STAT protein undergoes the process of acetylation, it can reduce the expression level of SHP protein^10,37^. Recent studies have shown that SMAD3 and STAT3 cooperative positively to enhance epithelial–mesenchymal transition (EMT) via SNAIL upregulation^52^. EMT is an evolutionary process that confers invasion features upon tumor cells by changes in cellular phenotype^52,53^. It is worth noting that in LIF signaling cascade, SMAD7 upregulation and positive cooperation of SMAD3-STAT3 are crosstalk points between LIF and TGFβ pathways which are considered in our model. To the best of our knowledge, it is the first dynamic model which describes the latter crosstalk point in LIF and TGFβ pathways. Moreover, in order to make a comprehensive understanding the roles of TGFβ, LIF, and CXCL12 pathways in malignant tumors, the interactions mentioned above are mathematically illustrated in their dynamical model.

The dynamical systems unfolding the biochemical interactions in CAFs and cancer cells are based on nonlinear ODEs which characterize concentration changes of the involved molecules over time. Biochemical components of signaling pathways and their corresponding states in ODEs for cancer cells and CAFs are shown in Tables S1 and S2, respectively. The ODEs are extracted from laws of mass action and Michaelis–Menten which are used vastly to analyze reaction kinetics^54,55^. Mathematical representation of biochemical reactions involved in cancer cell and CAF dynamics are shown in Tables 1 and 2. Mathematical expression of a biochemical interaction includes multiple parameters as shown in these two tables. In Tables S3 and S4, model parameters, values and their references are provided. The values of kinetic parameters are gathered from literature whenever possible. Due to the large number of parameters and unavailability of their exact values in previous studies, most of the parameters in Tables S3 and S4 were estimated using HUKF approach. HUKF is an accurate and robust method designed for state and also parameter estimation of nonlinear systems that use discrete time data points as observed measurements. This algorithm relies on transformed statistics of parameters which are propagated through main steps including prediction and correction. In the first step, using model structure, the desired parameters are estimated and then priory estimates are optimized following the second step. Both model and observed data uncertainties are taken into account by the Kalman filtering approach which leads to reliable estimations^29,56^. In this study, we used two different gene expression profiling results datasets of time course studies (GSE17708 and GSE129189 for cancer cell and CAF, respectively) from Gene Expression Omnibus (GEO) database as observation data for HUKF algorithm^57,58^. Using these tables, cancer cell and CAF dynamics are modeled as state space systems of 21 and 26 nonlinear ODEs, respectively which can be found in Supplementary materials.

**Table 1:** Biochemical reactions and their mathematical representations in cancer cell dynamics.

Description of the interaction	Mathematical expression
Production of TGFβ receptor	$v_{1} = k_{1}^{ + } x_{1}$
Degradation of TGFβ receptor	$v_{2} = k_{1}^{ - } x_{1}$
Association of TGFβ-TGFβR complex	$v_{3} = k_{2}^{ + } xu_{3} x_{1}^{2}$
Dissociation of TGFβ-receptor complex	$v_{4} = k_{2}^{ - } x_{2}$
Production of cytoplasmic SMAD3	$v_{5} = k_{3}^{ + } x_{3}$
Degradation of cytoplasmic SMAD3	$v_{6} = k_{3}^{ - } x_{3}$
Production of cytoplasmic SMAD4	$v_{7} = k_{4}^{ + } x_{5}$
Degradation of cytoplasmic SMAD4	$v_{8} = k_{4}^{ - } x_{5}$
Phosphorylation of SMAD3	$v_{9} = k_{5}^{ + } x_{2} \frac{{x_{3} }}{{x_{3} + K_{s1} }}$
Dephosphorylation of SMAD3	$v_{10} = k_{5}^{ - } x_{4}$
Association of pSMAD3-4 Complex	$v_{11} = k_{6}^{ + } x_{4} x_{5}$
Dissociation of pSMAD3-4 Complex	$v_{12} = k_{6}^{ - } x_{6}$
Nuclear import of pSMAD3-4 Complex	$v_{13} = k_{i7} x_{6}$
Nuclear export of pSMAD3-4 Complex	$v_{14} = k_{e7} x_{7}$
Degradation of pSMAD3-4 Complex	$v_{15} = k_{7}^{ - } x_{7}$
Production of SMAD7 in the pathway	$v_{16} = k_{8}^{ + } x_{7}$
Degradation of SMAD7	$v_{17} = k_{8}^{ - } x_{8}$
Inhibitory effect of SMAD7 on pSMAD3	$v_{18} = k_{8i}^{ - } x_{8}$
Production of TGFβ in the pathway	$v_{19} = k_{9}^{ + } x_{7}$
Degradation of TGFβ	$v_{20} = k_{9}^{ - } xu_{1}$
Association of TGFβ-TGFβR complex	$v_{3}^{^{\prime}} = k_{2}^{ + } xu_{1} x_{1}^{2}$
Production of LIF in the pathway	$v_{21} = k_{10}^{ + } x_{7}$
Degradation of LIF	$v_{22} = k_{10}^{ - } xu_{2}$
Production of CXCR4 in the pathway	$v_{23} = k_{11}^{ + } x_{7}$
Degradation of CXCR4	$v_{24} = k_{11}^{ - } x_{9}$

**Table 2:** Biochemical reactions and their mathematical representations in CAF dynamics.

Description of the interaction	Mathematical expression
Production of CXCL12 in the pathway	$v_{23} = k_{11}^{ + } x_{7}$
Degradation of CXCL12	$v_{24} = k_{11}^{ - } xu_{4}$
Production of LIF receptor	$w_{1} = h_{1}^{ + } x_{10}$
Degradation of LIF receptor	$w_{2} = h_{1}^{ - } x_{10}$
Association of LIF-LIFreceptor complex	$w_{3} = h_{2}^{ + } x_{10} xu_{2}$
Association of LIFcaf-LIFreceptor complex	$w_{3} = h_{2}^{ + } x_{10} x_{9}$
Dissociation of LIF-LIFreceptor complex	$w_{4} = h_{2}^{ - } x_{11}$
Production of JAK	$w_{5} = h_{3}^{ + } x_{12}$
Degradation of JAK	$w_{6} = h_{3}^{ - } x_{12}$
Phosphorylation of JAK by LIF-receptor complex	$w_{7} = h_{4}^{ + } x_{11} \frac{{x_{12} }}{{x_{12} + K_{s2} }}$
Dephosphorylation of Pjak	$w_{8} = h_{4}^{ - } x_{13}$
Production of STAT	$w_{9} = h_{5}^{ + } x_{14}$
Degradation of STAT	$w_{10} = h_{5}^{ - } x_{14}$
Phosphorylation of STAT by pJAK	$w_{11} = h_{6}^{ + } \frac{{x_{14} }}{{x_{14} + K_{s3} }}$
Dephosphorylation of pSTAT	$w_{12} = h_{6}^{ - } x_{15}$
Acetylation of pSTATn	$w_{13} = h_{7}^{ + } x_{16}$
Deacetylation of pSTATn	$w_{14} = h_{7}^{ - } x_{17}$
Inhibitory effect of pSTATnac on SHP1	$w_{15} = h_{7i}^{ - } x_{17}$
Nuclear import of pSTAT	$w_{16} = h_{i8} x_{15}$
Nuclear export of pSTAT	$w_{17} = h_{e8} x_{16}$
Production of SOCS3 downstream the pathway	$w_{18} = h_{9}^{ + } x_{16}$
Degradation of SOCS3	$w_{19} = h_{9}^{ - } x_{18}$
Production of SMAD7 downstream the pathway	$w_{20} = h_{10}^{ + } x_{16}$
Inhibitory effect of SOCS3 on STAT phosphorylation	$w_{22} = h_{10i}^{ - } x_{18}$
Production of SHP1	$w_{23} = h_{11}^{ + } x_{19}$
Degradation of SHP1	$w_{24} = h_{11}^{ - } x_{19}$
Inhibitory effect of SHP1 on STAT phosphorylation	$w_{25} = h_{11i}^{ - } x_{19}$
pSMAD3-pSTAT binding	$z_{1} = g_{1}^{ + } x_{4} x_{15}$
pSMAD3-pSTAT unbinding	$z_{2} = g_{1}^{ - } x_{20}$
Translocation of pSMAD3-pSTAT to nucleus	$z_{3} = g_{i} x_{20}$
Translocation of pSMAD3-pSTAT to cytoplasm	$z_{4} = g_{e} x_{21}$
Production of SNAIL in the pathway	$z_{5} = g_{2}^{ + } x_{21}$
Degradation of SNAIL	$z_{6} = g_{2}^{ - } x_{22}$

### Mathematical representation of intercellular communications in the TME

**Figure 4:**
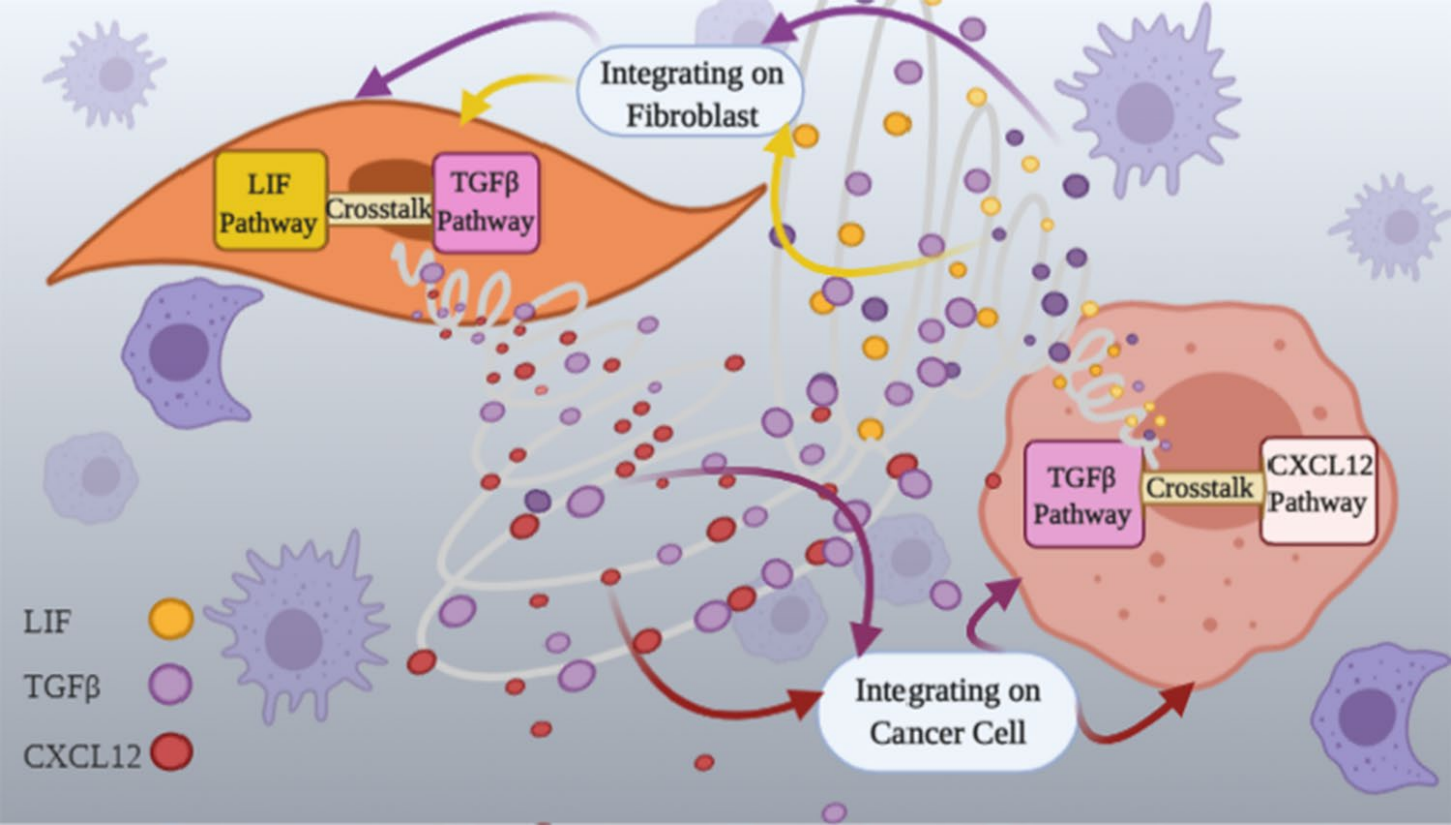
Intercellular interactions between CAFs and cancer cells in the TME. TGFβ, LIF and, CXCL12 pathways are responsible for the release of communicating signals in the TME. TGFβ and LIF molecules are circulating from cancer cells to CAFs, as well as CXCL12 and TGFβ from CAFs to cancer cells.

The intercellular scale is defined by the communication signals exchanged between cancer cells and CAFs which allow for the description of cancer cells’ effecs on CAF functioning and consequently, the impact of CAFs on cancer cell invasion. The basic structure of the model presented in this section is inspired by our previous work in^29^ with the notable exception that here we consider both spatial and temporal changes of signaling molecules in the model. As depicted in Fig. 4, TGFβ and LIF molecules transmit from cancer cells to CAFs where they stimulate the corresponding signaling pathways and lead to expression of metastatic factors such as CXCL12, SNAIL, TGFβ and LIF. Among these molecules, CXCL12 and TGFβ were identified as molecules that communicate signals from CAFs to cancer cells. They circulate in TME and once they reach the surface of cancer cells, tend to induce signal transduction.

To come up with a compendious view of signal transmission through the TME, we consider a three-dimensional space as tumor volume and its microenvironment. The mathematical representation for each signal is then provided using PDEs. Since gene expression of the signals, as well as their other regulatory activities occur within the cells, a number of terms in ODE systems of cancer cell and CAF will appear in PDEs.

On the other hand, when the transmission molecules circulating in the TME come into contact with the target cell, they are integrated onto the cell surface. The amount of reached factors on the cell is computationally modeled by an integral over the cell surface area. As a result, we have a coupled ODE–PDE framework which describes how CAFs and cancer cells interact in TME.

As it is shown in Table S1 and S2, our model includes four signaling molecules, $xu_{1} - xu_{4}$. Spatial–temporal changes of the $i$ th molecule ($u_{i}$) is modeled using reaction–diffusion equation in (1):

1 $$\partial_{t} u_{i} \left( {t,\vec{r}} \right) = D_{i} \Delta u_{i} \left( {t,\vec{r}} \right) - f_{i} \left( {u_{i},u_{j} } \right) \in \Omega \times \left[ {kT_{s}\, \left( {k + 1} \right)T_{s} } \right]\ ,k\epsilon N$$

where $\partial_{t}$ and $\Delta$ indicate first time derivative and Laplacian of diffusing signal ($u_{i}$)‚ respectively. Also, $\vec{r}$ is space vector $\vec{r} = \left( {xyz} \right) \in \Omega$ and $f_{i}$ denotes the kinetics of $u_{i}$ interactions with other factors ($u_{j}$) in domain $\Omega$ and time duration of $\left[ {kT_{s} \left( {k + 1} \right)T_{s} } \right]$ where $T_{s}$ is the simulation step and is defined later.${ }D_{i}$ is a positive constant which represents diffusion coefficient of $u_{i}$. The values of $D_{i}$ for each of the signals are shown in Table S5. In our model, $\Omega$ is assumed to be a sphere with a radius of 2 cm^59,60^ showing a three-dimensional space for the tumor. The Eq. (3) in Supplementary files shows the PDEs and complete form of $f_{i}$ dynamics for each signaling molecules, as well as their initial concentration. When solving a PDE, an initial condition and a boundary condition are typically needed, both of them are determined by the problem's time and space situations. We defined a constant value for initial condition ($\hat{u}_{0}$) of each signal at the first step of simulation, [0,$T_{s}$]. Then, at the end of each cycle, $\hat{u}_{0}$ is updated and used for the next step. In addition, boundary condition on $\Omega$ which shows the physical nature of signaling molecules on TME boundaries is considered as Dirichlet boundary condition.

As it is indicated in Fig. 4, signaling factors are integrated on each cell surface once they reached them and thus initiate the biological pathways within the cells. For determining the concentration level of molecules that is integrated ($\overline{u}_{i}$) at the exact part of the domain ($A$), we use the integral relation of (2):

2 $$\overline{u}_{i} = \int\limits_{A} {u_{i} (t,s)ds}$$

In the next step of the intercellular level, (1) and (2) are used for describing the distribution of signaling molecules in TME. Then, with the exception of ODEs describing the signaling factors variations, we incorporate the obtained four PDE-integral systems for $xu_{1}$ to $xu_{4}$ with the previous dynamical systems. Eventually, a coupled ODE–PDE dynamical system emerges that is able to mathematically explain the conversations among a CAF and a cancer cell, as well as their intracellular dynamics at the intercellular level.

### Biomechanical modeling movement of cancer cells in the TME

Cancer cells movement is occurred when they are prepared to detach from the tumor tissue and then leave the primary tumor under the influence of various intracellular and extracellular signal transduction pathways, as well as other TME components such as CAFs and ECM. ECM is one of the main TME components that is involved in metastasis. During the progression of metastasis, ECM is remodeled through a series of quantitative and qualitative modifications by degrading enzymes such as MMPs released by cancer cells^61^. So, in order to get a comprehensive view of cancer cells’ migration, we used an ODE adapted from^62^ that included ECM generation and degradation to model the function of ECM in the TME as (3):

3 $$\frac{d}{dt}\left[ {ECM} \right] = a_{1} \left[ {ECM} \right] - a_{2} \left[ {MMP} \right]\left[ {ECM} \right]$$

where $\left[ {ECM} \right]$ and $\left[ {MMP} \right]$ are ECM and MMP concentrations, and $a_{1}$ and $a_{2}$ are generation and degradation coefficients of ECM, respectively.

We upgraded the model of intercellular interactions between one cancer cell and CAF to a population level as a necessary step before mathematical modeling of metastasis. Then, the status of each cancer cell was assessed by tracking each cell individually. In this case, each cancer cell is thought to be able to choose between quiescent, proliferative, or migratory states with regards to the concentration levels of certain effective biochemical factors including SNAIL, ECM and CXCL12. These factors are chosen because of their key roles in EMT, cancer cell adherence to the primary tumor, and tumor cells growth and proliferation, respectively^53,63,64^. The variations of CXCL12 and ECM in intercellular level of our model have already been modeled, but concentration of SNAIL in the TME must also be considered. Thus, we used (4) to mathematically represent how SNAIL is diffused in the TME following its upregulation in CAFs.

4 $$\frac{d}{dt}\left[ {SNAIL\left( {t\ ,\vec{r}} \right)} \right] = D_{SNAIL} \Delta \left[ {SNAIL\left( {t\ ,\vec{r}} \right)} \right]$$

where $D_{SNAIL}$ is the diffusion coefficient of SNAIL and its value is shown in Table S5.

**Figure 5:**
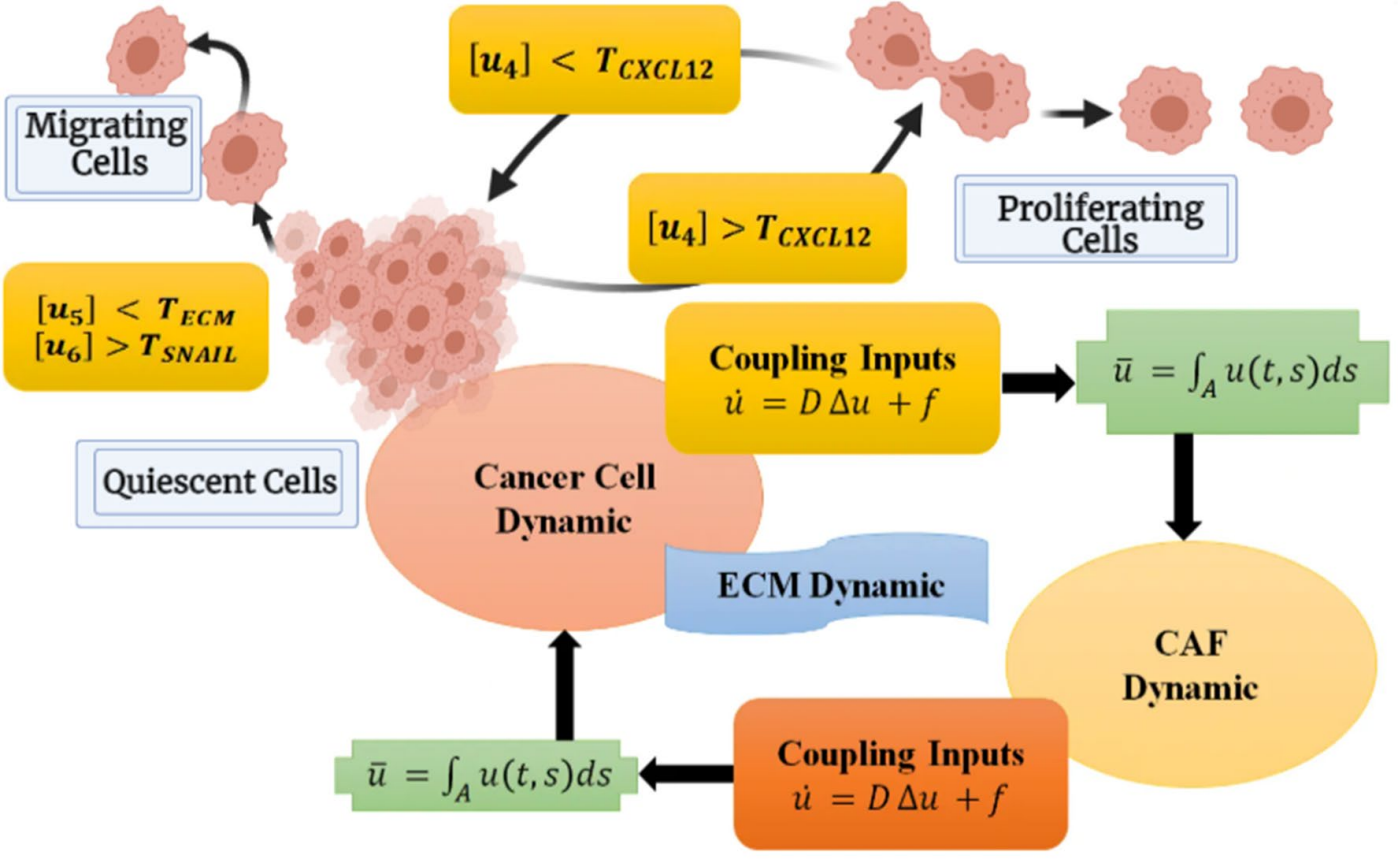
The multi-structure model for determining each cancer cell status. In this figure, u indicates the communicating signals which are transferred between cancer cells and CAFs in the tumor microenvironment (TGFβ and LIF molecules from cancer cells to CAFs, and CXCL12 and TGFβ from CAFs to cancer cells). Also, $u_{4}$, $u_{5}$, and $u_{6}$ represents CXCL12, ECM, and SNAIL concentration in the model. Furthermore, letter A indicates the surface area of a cancer cell or CAF which received a transduction signal and T implies the threshold amount of each signal which is indexed.

As it is depicted in Fig. 5, the modeling framework for designating the status of cancer cells is based on the newly implemented coupled ODE–PDE dynamical system in the previous sections, as well as (3) and (4). During the $\left[ {kT_{s} \left( {k + 1} \right)T_{s} } \right],k\epsilon N$ time interval, the combined dynamical system is solved and the levels of ECM, SNAIL and CXCL12 factors are determined at each $kT_{s} ,k\epsilon N$. Then, they are compared to a certain threshold value, and each cancer cell decides whether to keep or change its condition.

After assessing the status of cancer cells, we used an intuitive force-based biomechanical model based on the equation of motion to model movement of cancer cells. Cancer cells in migratory state will move in the population due to forces exerted in the TME as a result of interactions with other cells and the environment. In our model, cancer cells and CAFs are represented by spheres with radii of 8 and 7 µm, respectively^65,66^ and it is assumed that the forces are applied to their centers. Also, the trajectory of each cell in the TME is specified by an equation of motion resulting in a Newtonian dynamic. The basic equation that governs the cancer cells movement is described as (5):

5 $$F^{rep} + F^{adh} + F^{hap}_{ } + F^{act}_{ } = F^{fric}_{ }$$

where $F^{rep}$ and $F^{adh}$ are repulsive, and adhesive forces between each two cancer cells, respectively. Also, $F^{hap}_{ }$ stands for haptotaxis force which is the directional motility of cells in response to gradients of adhesive surfaces such as ECM^67,68^. Moreover, $F^{act}_{ }$ denotes the active force that is driven by SNAIL concentration as the determining factor in metastasis. We also denote the friction between cancer cells, and the TME by $F^{fric}$^69^. Mathematical representations of these forces are shown in Table 3. More details about the constants and parameters of Table 3 can be found in Table S9. In each time interval $\left[ {kT_{s} \left( {k + 1} \right)T_{s} } \right],k\epsilon N$, the velocity of cancer cells and consequently their displacement are calculated using (5) and Newtonian dynamic ($\Delta r = V.\Delta t$).

**Table 3:** Mathematical representations of forces in the multiscale model.

Force	Mathematical representation	Reference
Repulsive force	$$F_{ }^{rep} = \frac{4}{3}\hat{E}_{i} \sqrt {\hat{R}_{i} } \delta_{ij}^{{{\raise0.7ex\hbox{$3$} \!\mathord{\left/ {\vphantom {3 2}}\right.\kern-\nulldelimiterspace} \!\lower0.7ex\hbox{$2$}}}}$$	^69^
Adhesive force	$F_{ }^{adh} = - \pi W\hat{R}$	^69^
Haptotaxis force	$F^{hap} = - \chi \nabla \left[ {ECM} \right]$	^13^
Active Force	$F_{ }^{act} = \eta \frac{{A^{n} }}{{A^{n} + A_{0}^{n} }}{ }\hat{n}$	^70^
Friction force	$F^{fric}_{ } = - \Gamma_{ }^{cs} V_{ }$	^18^

## Experimental approaches

### Cell preparation

MCF-7 cells were cultured in Dulbecco’s Modified Eagle Medium/Nutrient Mixture F-12 (DMEM/F12, Bio Idea; Iran) supplemented with 10% fetal bovine serum (FBS; Viva, Iran), 100 mg/ml streptomycin and 100 U/ml penicillin in 5% CO_2_ atmosphere at 37 °C. When cultured cells reached more than 90% of confluence degree, cell culture supernatants were collected and stored at − 80 °C.

To isolate breast cancer-associated fibroblasts (CAFs), breast tissues were minced in DMEM/F12 supplemented with 10% FBS, and 1% Penicillin/Streptomycin. The tissues were cut into small pieces (1–2 mm^3^) and cultured in the same media at 37 °C for about 2 weeks until Fibroblasts grew out of the tissue. Then, cells were removed and cultured in T25 flask at 37 °C in a humidified atmosphere with 5% CO_2_. When cancer-associated fibroblasts (CAFs) cells reached more than 90% of confluence, cell culture supernatant was collected and stored at − 80 °C.

### Experiment

The CAFs were divided into two groups: group 1 (control, no treatment), and group 2 (cells treated with culture supernatant of MCF-7 cells). The CAFs were then incubated with DMEM F12 medium containing 10% FBS (250 µl) and supernatant collected from MCF-7 cells (750 µl) in a ratio of 1: 3 for 24 h, 48 h and 72 h. Also, MCF-7 cells were divided into two groups: group 1 (control, no treatment), group 2 (cells treated with culture supernatant of CAFs). MCF-7 cells were incubated with DMEM F12 medium containing 10% FBS and supernatant collected from CAFs in a ratio of 1: 3 for 24 h, 48 h and 72 h.

#### Wound scratch assay

Briefly, the MCF-7 cells, and CAFs (1 × 10^6^ cells/well) were plated in 12-well plates for 48 h to a confluence of about 90%, then wounded by scratching with a p100 pipette tip. Then, the debris was removed and the cells were washed once with 1 mL of the growth medium to assure the edges of the scratch were smoothed by washing. As mentioned above, the cells were cultured for 24 h, 48 h and 72 h. After different time courses, cells were washed twice with PBS and the wound was observed under a microscope. Cells were photographed before and after 24, 48 and 72 h of incubation and images were analyzed with ImageJ software to calculate the area of each scratch. Images of each well at different times were compared to comprehend the effect of treatments on cell migration^71^.

#### RNA extraction and cDNA synthesis

As mentioned above, the cells were cultured for 24 h, 48 h and 72 h. After each time course, total RNA was extracted from CAFs, and MCF-7 cells using the BioFACT™ Total RNA Prep Kit (BioFACT, South Korea), according to the manufacturer’s recommendations for cultured cells. RNA quantity was assessed using NanoDrop spectrophotometer. The average RNA yield was 0.5 μg RNA per 10^6^ cells. RNA was subsequently reverse-transcribed to cDNA using the BioFact™ RT Series (BioFACT, South Korea) according to the manufacturer’s recommendations. Then, they were incubated for 60 min at 42 °C and were terminated the reaction for 5 min at 95 °C.

#### Real-time polymerase chain reaction (PCR)

Quantitative PCR was performed in a Rotor-Gene 6000 (Corbett Life Science, Sydney, Australia). For mRNA quantification, the 2X Real-Time PCR Master Mix (BioFACT™, High ROX, Korea) was used in combination with pre-designed primers for Leukemia inhibitory factor (LIF), Transforming growth factor beta (TGF-β), SMAD7, C-X-C motif chemokine 12 (CXCL12) and for the reference gene GAPDH as the internal control, were designed using the Allele ID software and BLAST (NCBI online server).

All real-time PCR reactions were performed in duplicate at a final volume of 10 μl per well using a qPCR Master mix (Jena Bioscience, GmbH) and a Rotor-Gene 6000 (Corbett Life Science, Sydney, Australia). The following thermal cycling conditions were applied: polymerase activation/denaturation at 95 °C for 15 min, and 45 amplification cycles at 95 °C for 20 s, 60 °C for 20 s and 72 °C for 30 s. The mRNA fold increase or fold decrease with respect to control well was determined and Relative quantification (ΔΔCT) method was employed. The relative quantification of mRNAs was calculated using the 2 ^−ΔΔct^ method and according to the formula normalization ratio (N.R.) = 2^−ΔΔct^.

### Ethics declaration

We confirmed that all experiments were performed in accordance with relevant guidelines and regulations. Cancer-associated fibroblasts (CAFs) were isolated from surgical specimens taken from the tissue of patients with breast cancer at Seyed-al-Shohada Hospital, Isfahan. A written informed consent was taken from all participants. This study was approved by Isfahan University of Medical Sciences (the registration number: IR.MUI.MED.REC.1398.727).

## Results

### Nonlinear ODE modeling of cancer cell and CAF dynamics explains SMAD7, TGFβ, LIF and CXCL12 gene expression profiles

**Figure 6:**
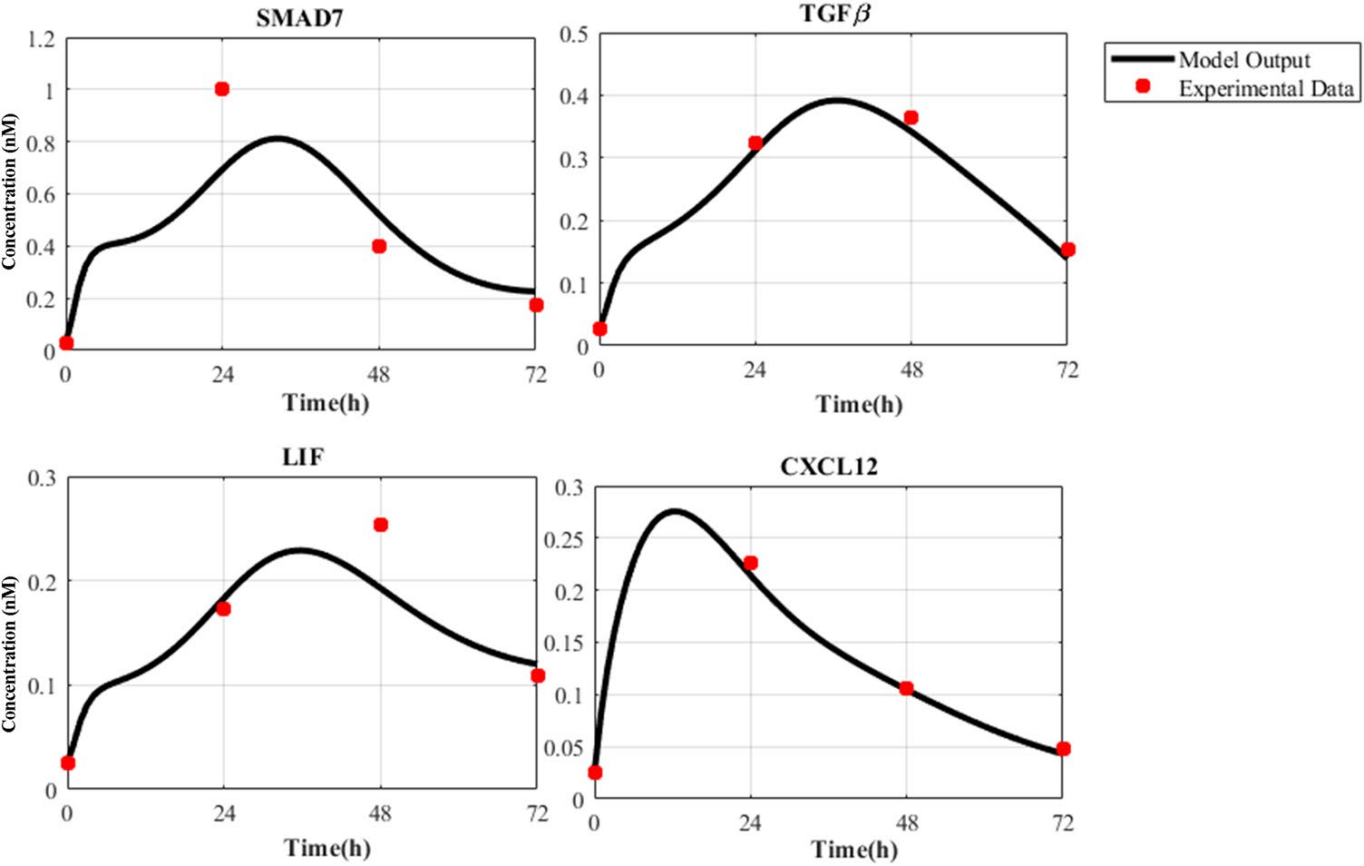
Experimental data and model outputs of ODE modeling of cancer cell. Gene expression profiles of SMAD7, TGFβ, LIF, and CXCL12 molecules in MCF-7 cell over four time points (0, 24, 48, and 72 h) and their corresponding behavior in the dynamic model are depicted by red dots and black curves, respectively.

**Figure 7:**
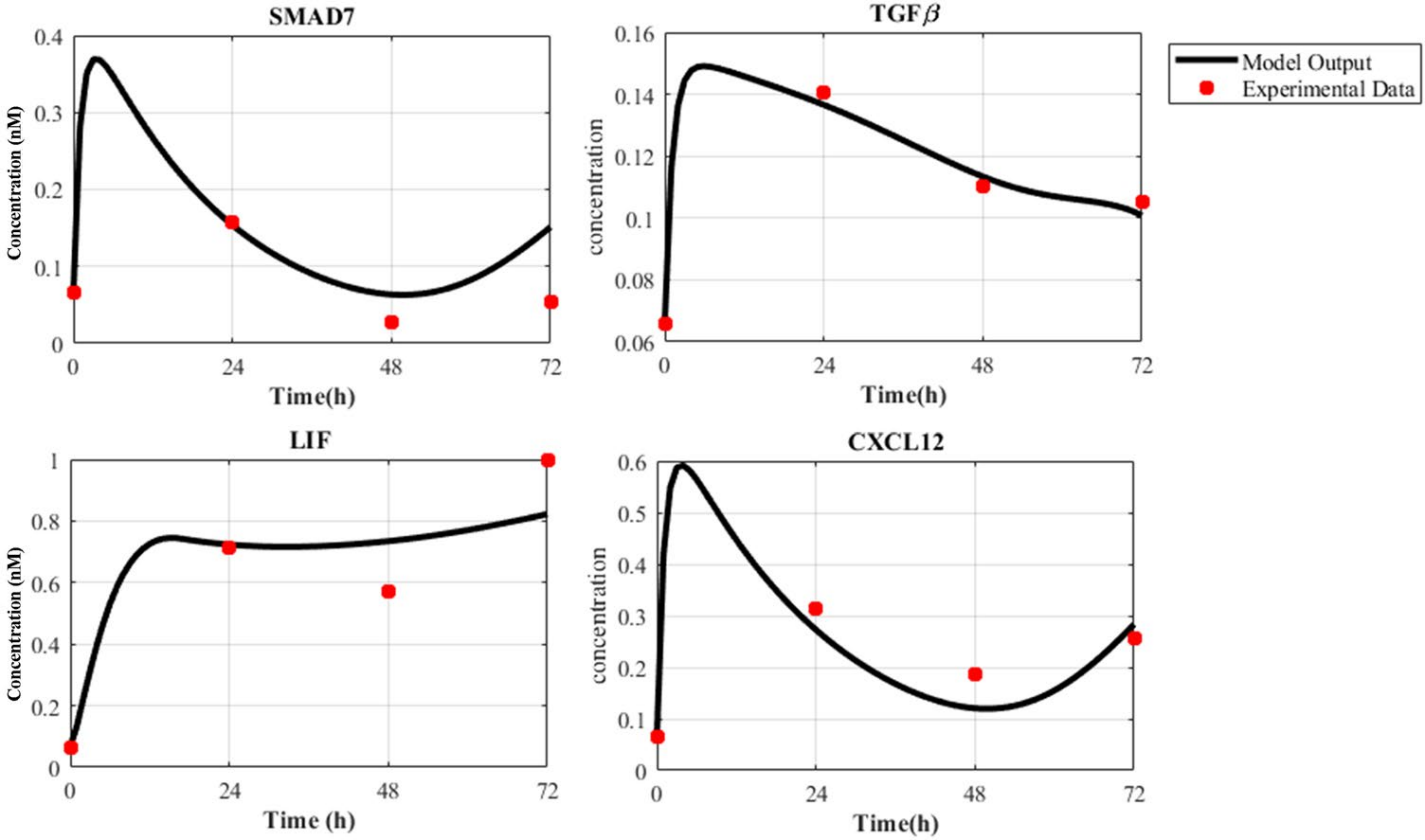
Experimental data and model outputs of ODE modeling of CAF. Gene expression profiles of SMAD7, TGFβ, LIF, and CXCL12 molecules in CAF over four time points (0, 24, 48, and 72 h) and their corresponding behavior in the dynamic model are depicted by red dots and black curves, respectively.

Mathematical modeling of intracellular interactions in cancer cells and CAFs allows determining gene expression variations of biochemical species involved in the reactions. The models presented in supplementary files were simulated using MATLAB ODE toolbox 2020a^72^. Figures 6 and 7 indicate the expression of fate-determining factors such as TGFβ, LIF, and CXCL12 increases in both cancer cells and CAF throughout the first three time periods. Also, simulation results succeeded to reproduce the behavior of SMAD7, TGFβ, LIF, and CXCL12 molecules in both cancer cell and CAF. This may be explained by looking at the goodness of fit criteria in Tables S5 and S6, which show how well our model outputs explain the variation in experimental data. Moreover, Bland–Altman graphs were provided for output genes as supplementary figures (Figs. S1 and S2) to highlight the agreement between two different datasets (model output and experimental data). Generally, the reported values and graphs for performance evaluation of output genes over four time points indicate a close enough proximity for model validation. Furthermore, using Morris method, we performed a global sensitivity analysis to evaluate the parameter effects on the model outputs^73^. Due to the large number of parameters, we selected a set of parameters which are directly affect the model outputs. In this method, selected parameters are changed over the entire range of probable values and the differences of the desired elements during these variations are calculated. Then for each parameter, mean and variance values are computed which are the indicators of effectiveness and nonlinearity of the parameter on the outputs, respectively. Sensitivity analysis showed that expression of TGFβ and its receptor, as well as expression of SMAD7 in this pathway are among the principle reactions that have the biggest effect on the outputs. More details on the selected parameters and also their mean and variance values obtained during the sensitivity analysis could be found in the Supplementary files.

### A force-based scheme incorporating the coupled ODE–PDE model and biomechanical forces describes cancer cells’ movement in population level

In order to investigate the cellular level of communication between cancer cells and CAFs, several PDEs are combined with ODE dynamics of each individual cell to model the interacting signals between cancer cells and CAFs circulating within the TME. The coupled ODE–PDE system is solved using finite element method (FEM) in MATLAB PDE toolbox 2020a. FEM is a numerical technique to solve PDEs over a defined space by dividing it into smaller parts^74^. The primary tumor in our model is a sphere with a radius of 2 cm that has been preprocessed by meshing to around 1000 subdomains. Also, the position of cancer cells and CAFs are randomly determined in the tumor. Then, using PDEs described as (1), TGFβ and LIF signals from cancer cells as well as TGFβ and CXCL12 molecules from CAFs release into the TME. Immediately after reaching these signals to the opposite cell type, signaling pathways within each cell get started according to the dynamics of cancer cell and CAF during a $\left[ {kT_{s} \left( {k + 1} \right)T_{s} } \right] ,k\epsilon N$ time interval. Furthermore, initial values of the next time step are updated in each $kT_{s}$. So, the cellular conversations between cancer cells and CAFs in a three dimensional domain are modeled using a coupled ODE–PDE dynamic system.

Then at the population level, the model has been developed to display movement of cancer cells as a result of their interactions with TME components and the designated status of each cell. In this scenario, cancer cells in migratory condition are under the influence of biomechanical forces that result from local interactions with neighbor cancer cells, CAFs and cancer cells. In summary, after solving coupled ODE–PDE dynamical system in a time interval, the level of diffused signals on each cell at $kT_{s} ,k\epsilon N$ are identified. Then, status of each cancer cell is determined in one of the quiescent, proliferating or migratory condition by the instruction illustrated in Fig. 5. Eventually, based on (5) and the defined forces in Table 3, displacements of cancer cells in migratory status are calculated. These events are repeated every $T_{s}$ seconds and at the end of each time step, initial values are updated for the next modeling step.

**Figure 8:**
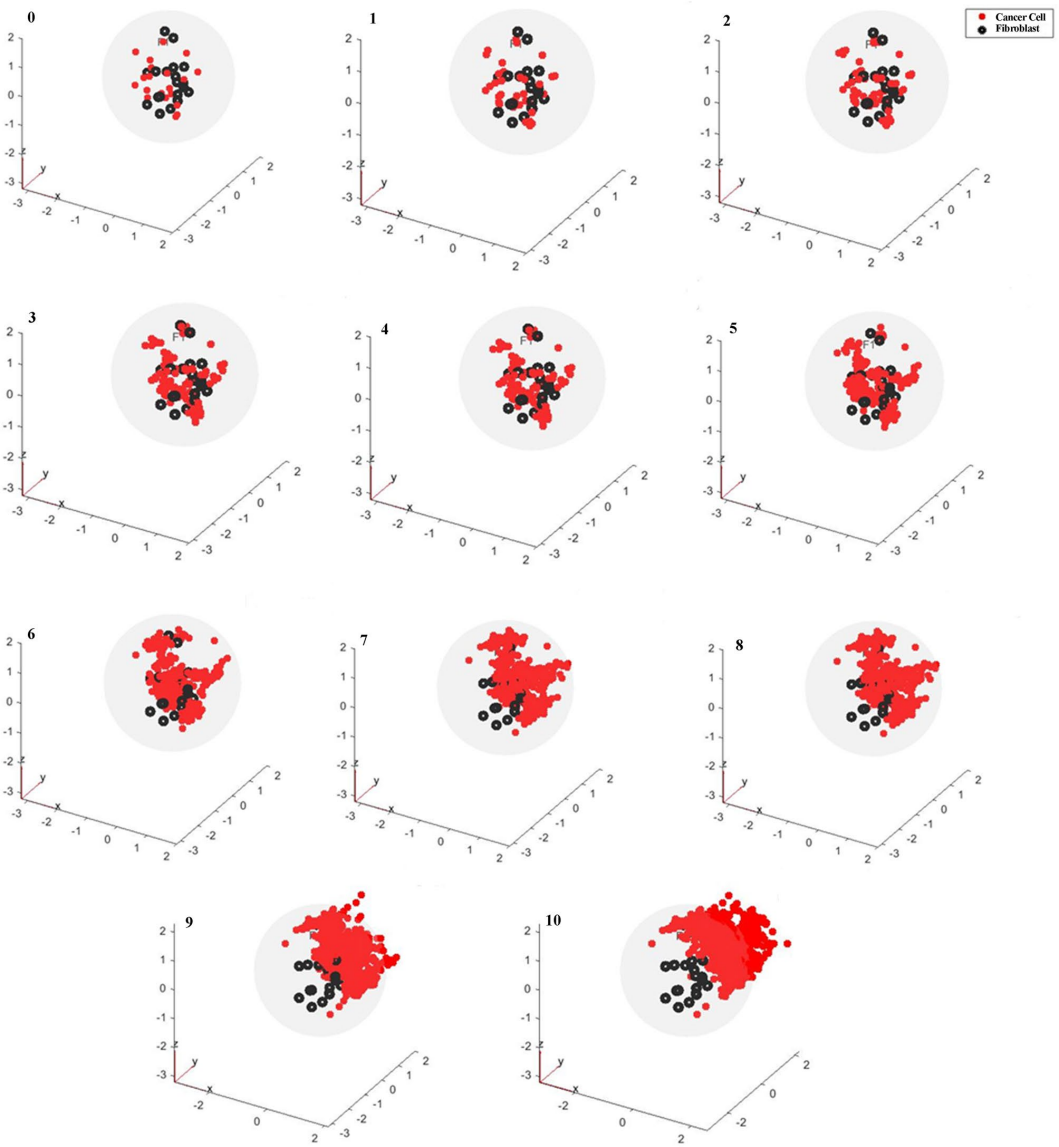
Simulation results of multiscale model of cancer cells movement during 10 time steps. Cancer cells and CAFs are depicted by red and black dots, respectively. Primary arrangement of cancer cells is randomly selected and it is shown in k = 0. After 10 repetitions, not only has the number of cells increased, but also they have been moved in space and left the primary tumor. The x–y-z axes have been scaled in centimeters.

Starting with 20 CAFs and 20 cancer cells randomly placed in a sphere as the primary tumor, this multiscale model is simulated using MATLAB 2020a. The process is repeated every $T_{s} = 0.05s$ and the results are depicted in Fig. 8. Initial cancer cells in the TME are considered to be in quiescent state and their primary arrangement is randomly selected. To validate the model, a wound scratch assay has been performed to evaluate MCF-7 cells migration while they are co-cultured with CAFs and the movement are assessed using Image J software. Comparison of the average displacement in wound scratch assay and model outputs are shown in Table 4, and the performance evaluation criteria in Table S8, as well as Bland–Altman graph in Fig. S3, indicate a proper performance of the applied techniques to model cancer cell metastasis.

**Table 4:** Average displacement of cancer cells in wound scratch assay versus model outputs.

Time (hour)	Displacement (mm) in wound scratch assay	Displacement (mm) in the model
0	0	0
24	0.158	0.180
48	0.223	0.264
72	0.672	0.634

## Discussion

It is well understood that cancer cells’ invasive features are highly dependent on their interactions with TME components, and thorough understanding of the communications could contribute to the design of new therapeutic approaches for preventing metastatic progression. Biological pathways, transmission signals, and mechanical forces occurring at different physical scales all influence the migration of cancer cells. In this paper, we have presented a multiscale model of metastasis with an emphasis on the impact of TME components such as CAFs and ECM. The model consists of three principle regulatory scales including intracellular, intercellular, and population levels which are validated against experimental data.

In the first step, biological pathways within the cancer cells and CAFs are modeled using ODE-based dynamical systems. TGFβ and CXCL12 pathways, as well as their crosstalk have been considered as the main regulatory mechanisms of cancer cell dynamics. In addition, an ODE model of CAF dynamic is derived using the mathematical depiction of physiological events involved in TGFβ and LIF signaling pathways. Since there are no exact values for several number of kinetic parameters, they are estimated using the HUKF algorithm. Moreover, the dynamical models produced results that are very similar to the experimental data of SMAD7, CXCL12, LIF, and TGFβ gene expression profiles. In this study, two different sets of experimental data have been used for the procedure of parameter estimation and model validation. Despite the fact that model yielded results in a close agreement to experimental data, small number of data points could be considered as a limitation of the study. However, it is inevitable that mathematical modeling of biological phenomena might be compelled to apply the limited number of data points, due to this fact that determining a large number of experimental data points can be impractical or impossible for a variety of reasons. To ensure meaningful results, we used technical methods such as HUKF algorithm which considers the uncertainty not only in the model parameters but also in the measurement datasets.

In order to describe how LIF, CXCL12, and TGFβ molecules which are considered as communicating signals circulate within the TME, we have considered a system of PDEs in a three-dimensional domain. The PDE system depicts the concentration variations of signaling molecules over time and space. Then, ODE models of cancer cells and CAFs are coupled to these PDEs through the mathematical statements utilized for evaluating the amount of signals received on the cell surfaces. As a result, a coupled system will be developed that can demonstrate the conversations of CAFs with cancer cells as well as their effect on tumor progression and invasion. Furthermore, by combination of coupled ODE–PDE modeling framework with ECM characteristics, the model succeeded to determine the status of each cancer cells in one of quiescent, proliferating or migratory conditions. Consequently, proliferating and migratory cells begin to replicate and migrate, causing tumor growth and invasion, respectively. Cancer cells’ movement are regulated by an equation of motion that includes adhesive, repulsive, haptotaxis, active, and friction forces applied to cancer cells. To demonstrate the functionality of the multiscale model, we performed a wound scratch assay test for measuring the displacement of MCF-7 cells co-cultured with CAFs at specific time points, and favorably acceptable accordance is observed when measured displacement in wound scratch assay and model outputs are compared. Although there are various methods of measuring directional collective cell migration, this assay was performed for measuring the tumor cells displacement due to its versatile application to fibroblasts and tumor cell types, simple and fast protocol, and easy analysis method. Moreover, not also cell migration, but also cell proliferation contribute to the wound healing, and these phenomena both has been considered in our modeling framework^96^.

Despite the fact that a number of experimental studies looked into the function of CAFs in malignant tumor metastasis, the mechanisms behind their influences on the invasiveness of cancer cells remained elusive, especially in the case of their mutual interactions in the TME. The proposed mathematical model is able to provide a comprehensive framework to illustrate not only the internal dynamics of CAFs and cancer cells, but also their interactive conversations throughout the TME. Furthermore, regulatory feedback loops that play a vital role in cancer cell and CAF phenotype change have been considered in the current study, which have not been investigated quantitatively so far. The model also shed more light on motility of cancer cells by taking into account ECM remodeling and its critical features in EMT. ECM as a TME component provides structural support to surrounding cells, but during advanced stages of metastasis, it undergoes some modifications and fails to keep cancer cells adhered together. As a result, cancer cells detach from the tumor tissue and leave the primary tumor. Moreover, the ability of the model to designate the cancer cells states makes an alive structure that is proliferating and moving, as well as allowing us to track the behavior of each cell during the simulation. Therefore, considering the fact that findings of the current multiscale model have been confirmed by experimental evidence, this paper can be regarded as a pioneer in the study of complicated phenomena such as tumor metastasis. From both empirical and computational point of view, our approaches pave the way for further studies and designing more therapeutic methods in cancer cell metastasis.

## Supplementary Information

Supplementary Information.

## Data Availability

The experimental datasets generated and analyzed by real time PCR and wound scratch assay during the current study, as well as simulation files are available from the corresponding author on reasonable request. All other relevant data related to the mathematical modeling are within the manuscript and its Supplementary files.

## References

[1] Micalizzi DS, Haber DA, Maheswaran S (2017). Cancer metastasis through the prism of epithelial-to-mesenchymal transition in circulating tumor cells. Mol. Oncol..

[2] Paul CD, Mistriotis P, Konstantopoulos K (2017). Cancer cell motility: Lessons from migration in confined spaces. Nat. Rev. Cancer.

[3] Clark AG, Vignjevic DM (2015). Modes of cancer cell invasion and the role of the microenvironment. Curr. Opin. Cell Biol..

[4] Maishi N, Hida K (2017). Tumor endothelial cells accelerate tumor metastasis. Cancer Sci..

[5] Wang M (2017). Role of tumor microenvironment in tumorigenesis. J. Cancer.

[6] Kharaishvili G (2014). The role of cancer-associated fibroblasts, solid stress and other microenvironmental factors in tumor progression and therapy resistance. Cancer Cell Int..

[7] Hanahan D, Coussens LM (2012). Accessories to the crime: Functions of cells recruited to the tumor microenvironment. Cancer Cell.

[8] Pietras K, Östman A (2010). Hallmarks of cancer: Interactions with the tumor stroma. Exp. Cell Res..

[9] Sewell-Loftin MK (2017). Cancer-associated fibroblasts support vascular growth through mechanical force. Sci. Rep..

[10] Kuzet S-E, Gaggioli C (2016). Fibroblast activation in cancer: When seed fertilizes soil. Cell Tissue Res..

[11] Deisboeck TS (2011). Multiscale cancer modeling. Annu. Rev. Biomed. Eng..

[12] Cai Y, Zhang J, Li Z (2016). Multi-scale mathematical modelling of tumour growth and microenvironments in anti-angiogenic therapy. Biomed. Eng. Online.

[13] Rocha H (2018). A hybrid three-scale model of tumor growth. Math. Mod. Methods Appl. Sci..

[14] Paterson C, Clevers H, Bozic I (2020). Mathematical model of colorectal cancer initiation. Proc. Natl. Acad. Sci..

[15] Watanabe Y (2016). A mathematical model of tumor growth and its response to single irradiation. Theor. Biol. Med. Model..

[16] Poleszczuk, J., Macklin, P. & Enderling, H. Agent-based modeling of cancer stem cell driven solid tumor growth, in *Stem Cell Heterogeneity*. (Springer, 2016). 335–346. 10.1007/7651_2016_346 PMC6587968 27044046

[17] Rahman MM (2017). A fully coupled space–time multiscale modeling framework for predicting tumor growth. Comput. Methods Appl. Mech. Eng..

[18] Ramis-Conde I (2009). Multi-scale modelling of cancer cell intravasation: The role of cadherins in metastasis. Phys. Biol..

[19] Andasari V (2011). Mathematical modeling of cancer cell invasion of tissue: Biological insight from mathematical analysis and computational simulation. J. Math. Biol..

[20] Nguyen Edalgo YT, Ford Versypt AN (2018). Mathematical modeling of metastatic cancer migration through a remodeling extracellular matrix. Processes.

[21] Nguyen Edalgo YT, Zornes AL, Ford Versypt AN (2019). A hybrid discrete–continuous model of metastatic cancer cell migration through a remodeling extracellular matrix. AIChE J..

[22] Kim Y, Othmer HG (2013). A hybrid model of tumor–stromal interactions in breast cancer. Bull. Math. Biol..

[23] Uppal A (2014). Investigation of the essential role of platelet-tumor cell interactions in metastasis progression using an agent-based model. Theor. Biol. Med. Model..

[24] Sfakianakis N (2017). A multiscale approach to the migration of cancer stem cells: Mathematical modelling and simulations. Bull. Math. Biol..

[25] Zhang L (2009). Simulating brain tumor heterogeneity with a multiscale agent-based model: Linking molecular signatures, phenotypes and expansion rate. Math. Comput. Model..

[26] Norton K-A, Popel AS (2014). An agent-based model of cancer stem cell initiated avascular tumour growth and metastasis: the effect of seeding frequency and location. J. R. Soc. Interface.

[27] Wadlow RC (2009). Systems-level modeling of cancer-fibroblast interaction. PLoS ONE.

[28] Kim Y (2010). Transformed epithelial cells and fibroblasts/myofibroblasts interaction in breast tumor: A mathematical model and experiments. J. Math. Biol..

[29] Heidary Z (2020). The double-edged sword role of fibroblasts in the interaction with cancer cells; an agent-based modeling approach. PLoS ONE.

[30] Meskin N (2013). Parameter estimation of biological phenomena: An unscented Kalman filter approach. IEEE/ACM Trans. Comput. Biol. Bioinf..

[31] Wang SE (2009). A mathematical model quantifies proliferation and motility effects of TGF-β on cancer cells. Comput. Math. Methods Med..

[32] Bellomo C, Caja L, Moustakas A (2016). Transforming growth factor β as regulator of cancer stemness and metastasis. Br. J. Cancer.

[33] Sakaki-Yumoto M, Katsuno Y, Derynck R (2013). TGF-β family signaling in stem cells. Biochim. Biophys. Acta (BBA)-Gen. Subj..

[34] Shi Y, Riese DJ, Shen J (2020). The role of the CXCL12/CXCR4/CXCR7 chemokine axis in cancer. Front. Pharmacol..

[35] Yue, X., Wu, L. & Hu, W. The regulation of leukemia inhibitory factor*.**Cancer Cell Microenviron.*, **2**(3) (2015). 10.14800/ccm.877 PMC4722946 26807429

[36] Albrengues J (2014). LIF mediates proinvasive activation of stromal fibroblasts in cancer. Cell Rep..

[37] Albrengues J (2015). Epigenetic switch drives the conversion of fibroblasts into proinvasive cancer-associated fibroblasts. Nat. Commun..

[38] Drabsch Y, Ten Dijke P (2012). TGF-β signalling and its role in cancer progression and metastasis. Cancer Metastasis Rev..

[39] Syed V (2016). TGF-β signaling in cancer. J. Cell. Biochem..

[40] Karagiannis GS (2012). Cancer-associated fibroblasts drive the progression of metastasis through both paracrine and mechanical pressure on cancer tissue. Mol. Cancer Res..

[41] Righetti, A., *et al*. CXCL12 and its isoforms: different roles in pancreatic cancer? *J*. *Oncol*. 2019 (2019). 10.1155/2019/9681698 PMC6582792 31275385

[42] Kojima Y (2010). Autocrine TGF-β and stromal cell-derived factor-1 (SDF-1) signaling drives the evolution of tumor-promoting mammary stromal myofibroblasts. Proc. Natl. Acad. Sci..

[43] Wang Y (2017). Stromal cell-derived factor-1α and transforming growth factor-β1 synergistically facilitate migration and chondrogenesis of synovium-derived stem cells through MAPK pathways. Am. J. Trans. Res..

[44] Chinni SR (2006). CXCL12/CXCR4 signaling activates Akt-1 and MMP-9 expression in prostate cancer cells: The role of bone microenvironment-associated CXCL12. Prostate.

[45] Schulze-Osthoff K (1997). Regulation of NF-κB activation by MAP kinase cascades. Immunobiology.

[46] Wu K-IS, Schmid-Schönbein GW (2011). Nuclear factor kappa B and matrix metalloproteinase induced receptor cleavage in the spontaneously hypertensive rat. Hypertension.

[47] Winkler J (2020). Concepts of extracellular matrix remodelling in tumour progression and metastasis. Nat. Commun..

[48] Paolillo M, Schinelli S (2019). Extracellular matrix alterations in metastatic processes. Int. J. Mol. Sci..

[49] Jenkins BJ (2005). Hyperactivation of Stat3 in gp130 mutant mice promotes gastric hyperproliferation and desensitizes TGF-β signaling. Nat. Med..

[50] To K (2004). Constitutional activation of IL-6-mediated JAK/STAT pathway through hypermethylation of SOCS-1 in human gastric cancer cell line. Br. J. Cancer.

[51] Babon, J. J., Varghese, L. N. & Nicola, N. A. Inhibition of IL-6 family cytokines by SOCS3. in *Seminars in immunology*. (Elsevier, 2014). 10.1016/j.smim.2013.12.004 PMC3970923 24418198

[52] Itoh Y, Saitoh M, Miyazawa K (2018). Smad3–STAT3 crosstalk in pathophysiological contexts. Acta Biochim. Biophys. Sin..

[53] Mittal V (2018). Epithelial mesenchymal transition in tumor metastasis. Annu. Rev. Pathol..

[54] Ingalls BP (2013). Mathematical Modeling in Systems Biology: An Introduction.

[55] Chen WW, Niepel M, Sorger PK (2010). Classic and contemporary approaches to modeling biochemical reactions. Genes Dev..

[56] Lillacci G, Khammash M (2010). Parameter estimation and model selection in computational biology. PLoS Comput. Biol..

[57] Sartor MA (2010). ConceptGen: A gene set enrichment and gene set relation mapping tool. Bioinformatics.

[58] Ershaid N (2019). NLRP3 inflammasome in fibroblasts links tissue damage with inflammation in breast cancer progression and metastasis. Nat. Commun..

[59] Iber, D. Numerical Solution of reaction-diffusion problems*.* Department for Biosystems Science and Engineering (D-BSSE), ETH Zurich, Swiss Institute of Bioinformatics.

[60] Friedmann, E. PDE/ODE modeling and simulation to determine the role of diffusion in long-term and -range cellular signaling. BMC Biophys. **8**(10) (2015). 10.1186/s13628-015-0024-8 PMC4606510 26473028

[61] Lu P (2011). Extracellular matrix degradation and remodeling in development and disease. Cold Spring Harb. Perspect. Biol..

[62] Kim Y, Friedman A (2010). Interaction of tumor with its micro-environment: A mathematical model. Bull. Math. Biol..

[63] Orimo A (2005). Stromal fibroblasts present in invasive human breast carcinomas promote tumor growth and angiogenesis through elevated SDF-1/CXCL12 secretion. Cell.

[64] Kai F, Drain AP, Weaver VM (2019). The extracellular matrix modulates the metastatic journey. Dev. Cell.

[65] Frisch T, Thoumine O (2002). Predicting the kinetics of cell spreading. J. Biomech..

[66] Moore, M. J., Strohm, E. M. & Kolios, M. C. Evaluation of the morphological parameters of cancer cells using high-frequency ultrasound and photoacoustics. in *2015 IEEE International Ultrasonics Symposium (IUS)*. 2015. IEEE.

[67] Oudin MJ (2016). Tumor cell–driven extracellular matrix remodeling drives haptotaxis during metastatic progression. Cancer Discov..

[68] Rikitake Y, Takai Y (2011). Directional cell migration: Regulation by small G proteins, Nectin-like molecule-5, and afadin. Int. Rev. Cell Mol. Biol..

[69] Van Liedekerke, P., Buttenschön, A. & Drasdo, D. Off-lattice agent-based models for cell and tumor growth: numerical methods, implementation, and applications, in *Numerical Methods and Advanced Simulation in Biomechanics and Biological Processes*. (Elsevier, 2018), 245–267.

[70] Cao Y, Ghabache E, Rappel WJ (2019). Plasticity of cell migration resulting from mechanochemical coupling. Elife.

[71] Rueden CT (2017). Image J2: ImageJ for the next generation of scientific image data. BMC Bioinf..

[72] The Math Works, I., *MATLAB. Version 2020a*. 2020, The Math Works, Inc. p. Computer Software.

[73] Jin, Y. *et al*. Improving data fitting of a signal transduction model by global sensitivity analysis. In *2007 American Control Conference*, IEEE, (2007), 2708–2713.

[74] Reddy JN (2019). Introduction to the Finite Element Method.

[75] Zi Z (2011). Quantitative analysis of transient and sustained transforming growth factor-β signaling dynamics. Mol. Syst. Biol..

[76] Cellière G (2011). The plasticity of TGF-β signaling. BMC Syst. Biol..

[77] Nicklas D, Saiz L (2013). Computational modelling of Smad-mediated negative feedback and crosstalk in the TGF-β superfamily network. J. R. Soc. Interface.

[78] Clarke DC, Betterton M, Liu X (2006). Systems theory of Smad signalling. IEE Proc.-Syst. Biol..

[79] Wegner K (2012). Dynamics and feedback loops in the transforming growth factor β signaling pathway. Biophys. Chem..

[80] Coggins NL (2014). CXCR7 controls competition for recruitment of β-arrestin 2 in cells expressing both CXCR4 and CXCR7. PLoS ONE.

[81] Chang, S.-W.L. *Mechanistic and Statistical Models to Understand CXCL12/CXCR4/CXCR7 in Breast Cancer*. (2015).

[82] Anderson MW (2019). Mathematical modeling highlights the complex role of AKT in TRAIL-induced apoptosis of colorectal carcinoma cells. Iscience.

[83] Pappalardo F (2016). Computational modeling of PI3K/AKT and MAPK signaling pathways in melanoma cancer. PLoS ONE.

[84] Lee T (2008). Sensing and integration of Erk and PI3K signals by Myc. PLoS Comput. Biol..

[85] Fujita KA (2010). Decoupling of receptor and downstream signals in the Akt pathway by its low-pass filter characteristics. Sci. Sign..

[86] Radulescu O (2008). Robust simplifications of multiscale biochemical networks. BMC Syst. Biol..

[87] Hui W (2016). Oxidative changes and signalling pathways are pivotal in initiating age-related changes in articular cartilage. Ann. Rheum. Dis..

[88] Singh A, Jayaraman A, Hahn J (2006). Modeling regulatory mechanisms in IL-6 signal transduction in hepatocytes. Biotechnol. Bioeng..

[89] Raia V (2011). Dynamic mathematical modeling of IL13-induced signaling in Hodgkin and primary mediastinal B-cell lymphoma allows prediction of therapeutic targets. Can. Res..

[90] Mayya V, Loew L (2005). STAT module can function as a biphasic amplitude filter. Syst. Biol..

[91] Yamada S (2003). Control mechanism of JAK/STAT signal transduction pathway. FEBS Lett..

[92] Qi YF (2013). Elucidating the crosstalk mechanism between IFN-gamma and IL-6 via mathematical modelling. BMC Bioinf..

[93] Goodhill GJ (1997). Diffusion in axon guidance. Eur. J. Neurosci..

[94] Szatmary AC, Stuelten CH, Nossal R (2014). Improving the design of the agarose spot assay for eukaryotic cell chemotaxis. RSC Adv..

[95] Dattoli AA (2015). Domain analysis of the Nematostella vectensis SNAIL ortholog reveals unique nucleolar localization that depends on the zinc-finger domains. Sci. Rep..

[96] Molinie N, Gautreau A (2018). Directional Collective Migration in Wound Healing Assays in Cell Migration.

