# Supplementary material for: Multiscale modeling of collective cell migration elucidates the mechanism underlying tumor–stromal interactions in different spatiotemporal scales
Source: Sci Rep. 2022 Sep 28;12:16242. doi: 10.1038/s41598-022-20634-5 (PMC9519582; doi:10.1038/s41598-022-20634-5)
Supplement: Supplementary file 1 — Supplementary Information. [file 41598_2022_20634_MOESM1_ESM.pdf]

**Multiscale modeling of collective cell migration elucidates the mechanism underlying tumor-stromal interactions in different spatiotemporal scales**

Zarifeh Heidary[1], Shaghayegh Haghjooy Javanmard[2], Iman Izadi[1], Nasrin Zare[3] and Jafar Ghaisari[1]*

1 Department of Electrical and Computer Engineering, Isfahan University of Technology, Isfahan, 84156-83111, Iran, 2 Department of Physiology, Applied Physiology Research Center, Isfahan Cardiovascular Research Institute, Isfahan University of Medical Sciences, Isfahan, 81746-73461, Iran, 3 [1]School of Medicine, Najafabad Branch, Islamic Azad University, Isfahan, Iran

# Supplementary Materials

Table S1: biochemical reactants in TGFβ and CXCL12 signaling pathways and their corresponding states in the dynamical model of cancer cells. It should be noted that $xu_1$ to $xu_4$ act as signals which initiate the signal transduction pathways.

| Components in the signaling pathway | State |
|---|---|
| TGFβ receptor | $x_1$ |
| TGFβ-TGFβ receptors Complex | $x_2$ |
| Cytoplasmic SMAD3 | $x_3$ |
| Cytoplasmic Phosphorylated SMAD3 | $x_4$ |
| SMAD4 | $x_5$ |
| Cytoplasmic SMAD3-SMAD4 Complex | $x_6$ |
| SMAD3-SMAD4 Complex in the nucleus | $x_7$ |
| SMAD7 | $x_8$ |
| CXCR4 | $x_9$ |
| CXCL12-CXCR4 Complex | $x_{10}$ |
| PI3K | $x_{11}$ |
| Activated PI3K | $x_{12}$ |
| Akt | $x_{13}$ |
| Phosphorylated Akt | $x_{14}$ |
| NFκB | $x_{15}$ |
| Activated NFκB | $x_{16}$ |
| MMP | $x_{17}$ |
| TGFβ (production of TGFβ pathway) | $xu_1$ |
| LIF (production of TGFβ pathway) | $xu_2$ |
| TGFβ (external signal) | $xu_3$ |
| CXCL12 (external signal) | $xu_4$ |

Table S2: biochemical reactants in TGFβ and LIF signaling pathways and their corresponding states in the dynamical model of CAFs. It should be noted that $xu_1$ to $xu_4$ act as signals which initiate the signal transduction pathways.

| Components in the signaling pathway | State |
|---|---|
| TGFβ receptor | $x_1$ |
| TGFβ-TGFβ receptors Complex | $x_2$ |
| SMAD3 | $x_3$ |
| Phosphorylated SMAD3 | $x_4$ |
| SMAD4 | $x_5$ |
| SMAD3-SMAD4 Complex | $x_6$ |
| SMAD3-SMAD4 Complex in the nucleus | $x_7$ |
| SMAD7 | $x_8$ |
| LIF | $x_9$ |
| LIF receptor | $x_{10}$ |

| | |
|---|---|
| LIF-LIF receptor complex | $x_{11}$ |
| JAK | $x_{12}$ |
| Phosphorylated JAK | $x_{13}$ |
| STAT | $x_{14}$ |
| Phosphorylated STAT | $x_{15}$ |
| Nuclear pSTAT | $x_{16}$ |
| Acetylated pSTAT | $x_{17}$ |
| SOCS | $x_{18}$ |
| SHP | $x_{19}$ |
| pSMAD3-pSTAT Complex | $x_{20}$ |
| pSMAD3-pSTAT Complex in the nucleus | $x_{21}$ |
| SNAIL | $x_{22}$ |
| TGFβ (external signal) | $xu_1$ |
| LIF (external signal) | $xu_2$ |
| TGFβ (production of TGFβ pathway) | $xu_3$ |
| CXCL12 (production of TGFβ pathway) | $xu_4$ |

Table S3: Parameters in the dynamical model of TGFβ and CXCL12 pathways within cancer cells

| **Description of the reaction** | **kinetic rate** | **Value** | **Reference** |
|---|---|---|---|
| Production rate of TGFβ receptor | ${k_1}^+$ | 0.13298 (1/min) | Estimated |
| Degradation rate of TGFβ receptor | ${k_1}^-$ | 0.003 (1/min) | [75] |
| Association rate of TGFβ-TGFβR complex | ${k_2}^+$ | 0.5 (1/ nMol $^2$.min) | Estimated |
| Dissociation rate of TGFβ-TGFβR complex | ${k_2}^-$ | 0.24 (1/min) | [76] |
| Production rate of cytoplasmic SMAD3 | ${k_3}^+$ | 0.152 (1/min) | Estimated |
| Degradation rate of cytoplasmic SMAD3 | ${k_3}^-$ | 0.000646 (1/min) | [77] |
| Production rate of cytoplasmic SMAD4 | ${k_4}^+$ | 0.12512 (1/min) | Estimated |
| Degradation rate of cytoplasmic SMAD4 | ${k_4}^-$ | 0.0012 (1/min) | [77] |
| Phosphorylation rate of SMAD3 | ${k_5}^+$ | 3.51 (1/min) | [78] |
| Dephosphorylation rate of pSMAD3 | ${k_5}^-$ | 0.00252 (1/min) | [77] |
| Association rate of pSMAD3-4 complex | ${k_6}^+$ | 0.02118 (1/nMol.min) | Estimated |
| Dissociation rate of pSMAD3-4 complex | ${k_6}^-$ | 0.54 (1/min) | [76] |
| Nuclear import rate of pSMAD3-4 complex | $k_{i7}$ | 0.16 (1/min) | [79] |
| Nuclear export rate of pSMAD3-4 complex | $k_{e7}$ | 0.25 (1/min) | [77] |
| Degradation rate of pSMAD3-4 complex | ${k_7}^-$ | 0.005 (1/min) | [79] |
| Production rate of SMAD7 in the pathway | ${k_8}^+$ | 0.48 (1/min) | Estimated |
| Degradation rate of SMAD7 | ${k_8}^-$ | 0.00483 (1/min) | [76] |
| Inhibitory effect of SMAD7 on pSMAD3 | ${k_{8i}}^-$ | 0.0421 (1/min) | Estimated |
| Production rate of TGFβ in the pathway | ${k_9}^+$ | 0.12 (1/min) | Estimated |
| Degradation rate of TGFβpr | ${k_9}^-$ | 0.0051 (1/min) | Estimated |
| Production rate of LIF in the pathway | ${k_{10}}^+$ | 0.091 (1/min) | Estimated |
| Degradation rate of LIFpr | ${k_{10}}^-$ | 0.029 (1/min) | Estimated |
| Production rate of CXCR in the pathway | ${k_{11}}^+$ | 0.93 (1/min) | Estimated |
| Degradation rate of CXCR | ${k_{11}}^-$ | 0.58 (1/min) | Estimated |
| Phosphorylation binding constant for SMAD3 | $K_{s1}$ | 1 (nMol) | Estimated |
| Association rate of CXCL12-CXCR4 complex | ${p_2}^+$ | 0.126 (1/nMol.min) | [80, 81] |
| Dissociation rate of CXCL12-CXCR4 complex | ${p_2}^-$ | 0.145 (1/min) | Estimated |
| Production rate of PI3K | ${p_3}^+$ | 0.0215 (1/min) | Estimated |
| Degradation rate of PI3K | ${p_3}^-$ | 0.012 (1/min) | Estimated |
| Production rate of Akt | ${p_4}^+$ | 0.022 (1/min) | Estimated |
| Degradation rate of Akt | ${p_4}^-$ | 0.0003474 (1/min) | [82] |

| | | | |
|---|---|---|---|
| Phosphorylation rate of PI3K by CC | ${p_5}^+$ | 0.015 (1/min) | Estimated |
| Dephosphorylation rate of PI3K | ${p_5}^-$ | 0.03 (1/min) | [83] |
| Phosphorylation rate of Akt by pPI3K | ${p_6}^+$ | 6 (1/min) | [84] |
| Dephosphorylation rate of Akt | ${p_6}^-$ | 1.98 (1/min) | [85] |
| Production rate of NFκB | ${p_7}^+$ | 0.02512 (1/min) | Estimated |
| Degradation rate of NFκB | ${p_7}^-$ | 0.012 (1/min) | [86] |
| Activation of NFκB by pAkt | ${p_8}^+$ | 1.2 (1/min) | Estimated |
| Deactivation of NFκB | ${p_8}^-$ | 0.6 (1/min) | [87] |
| Production of MMP by pNFκB | ${p_9}^+$ | 0.8326 (1/min) | Estimated |
| Degradation of MMP | ${p_9}^-$ | 0.000384 (1/min) | [87] |
| Degradation of CXCL12 | ${p_{10}}^-$ | 0.041 (1/min) | Estimated |
| Phosphorylation binding constant for PI3K | $K_{p1}$ | 10 (nMol) | Estimated |
| Phosphorylation binding constant for Akt | $K_{p2}$ | 10 (nMol) | [84] |
| Activation constant for NFκB | $K_{p3}$ | 100 (nMol) | Estimated |

Table S4: Parameters in the dynamical model of LIF pathway within CAFs. The parameters of TGFβ pathway in CAFs are the same as Table S3.

| **Description of the reaction** | **kinetic rate** | **Value** | **Reference** |
|---|---|---|---|
| Production rate of CXCL12 in pathway | ${k_{11}}^+$ | 0.861 (1/min) | Estimated |
| Degradation rate of CXCL12 | ${k_{11}}^-$ | 0.041 (1/min) | Estimated |
| Production rate of LIF receptor | $h_1^+$ | 0.19 (1/min) | Estimated |
| Degradation rate of LIF receptor | $h_1^-$ | 0.0968 (1/min) | Estimated |
| Association rate of LIF-LIF receptor complex | $h_2^+$ | 6 (1/nMol.min) | [88] |
| Dissociation rate of LIF-LIF receptor complex | $h_2^-$ | 0.491 (1/min) | Estimated |
| Production rate of JAK | $h_3^+$ | 0.026 (1/min) | Estimated |
| Degradation rate of JAK | $h_3^-$ | 0.0012 (1/min) | Estimated |
| Phosphorylation rate of JAK by LIF receptor | $h_4^+$ | 0.157 (1/min) | [89] |
| Dephosphorylation rate of pJAK | $h_4^-$ | 0.000621906 (1/min) | [89] |
| Production rate of STAT | $h_5^+$ | 0.21 (1/min) | Estimated |
| Degradation rate of STAT | $h_5^-$ | 0.02 (1/min) | Estimated |
| Phosphorylation rate of STAT by pJAK | $h_6^+$ | 60 (1/min) | [90] |
| Dephosphorylation rate of pSTAT | $h_6^-$ | 0.18 (1/min) | [91] |
| Acetylation rate of pSTAT | $h_7^+$ | 0.18 (1/min) | Estimated |
| Deacetylation rate of pSTAT | $h_7^-$ | 0.08 (1/min) | Estimated |
| Inhibitory effect of pSTATac on SHP1 | $h_{7i}^-$ | 0.1 (1/min) | Estimated |
| Nuclear import rate of pSTAT | $h_{i8}$ | 0.001 (1/min) | Estimated |
| Nuclear export rate of pSTAT | $h_{e8}$ | 3 (1/min) | [88, 92] |
| Production rate of SOCS in the pathway | $h_9^+$ | 0.6 (1/min) | [88, 91, 92] |
| Degradation rate of SOCS | $h_9^-$ | 0.03 (1/min) | [88, 91, 92] |
| Production rate of SMAD7 in the LIF pathway | $h_{10}^+$ | 0.6 (1/min) | [91] |
| Inhibitory effect of SOCS on STAT phosphorylation | $h_{10i}^-$ | 0.006 (1/min) | Estimated |
| Production rate of SHP1 | $h_{11}^+$ | 0.3215 (1/min) | Estimated |
| Degradation rate of SHP1 | $h_{11}^-$ | 0.213 (1/min) | Estimated |
| Inhibitory effect of SHP1 on STAT phosphorylation | $h_{11i}^-$ | 0.09 (1/min) | Estimated |
| Phosphorylation binding constant for JAK | $K_{s2}$ | 0.00005 (nMol) | Estimated |
| Acetylation binding constant for STAT | $K_{s3}$ | 4000 (nMol) | [90] |
| pSMAD3-pSTAT association rate | $g_1^+$ | 0.5 (1/nMol.min) | Estimated |
| pSMAD3-pSTAT unbinding rate | $g_1^-$ | 0.12 (1/min) | Estimated |
| Translocation of pSMAD3-pSTAT to nucleus | $g_i$ | 0.2 (1/min) | Estimated |
| Translocation of pSMAD3-pSTAT to cytoplasm | $g_e$ | 0.18 (1/min) | Estimated |

| | | | |
|---|---|---|---|
| Production rate of SNAIL in the pathway | $g_2^+$ | 0.31 (1/min) | Estimated |
| Degradation of SNAIL | $g_2^-$ | 0.05 (1/min) | Estimated |

Cancer cell dynamics is shown in (1) as follows:

$$\frac{d}{dt}x_1 = v_1 - v_2 - v_3 - v_3{}' + v_4$$
$$\frac{d}{dt}x_2 = v_3 - v_3{}' - v_4 - v_9$$
$$\frac{d}{dt}x_3 = v_5 - v_6 - v_9 + v_{10} + v_{18}$$
$$\frac{d}{dt}x_4 = v_9 - v_{10} - v_{11} + v_{12} - v_{18}$$
$$\frac{d}{dt}x_5 = v_7 - v_8 - v_{11} + v_{12}$$
$$\frac{d}{dt}x_6 = v_{11} - v_{12} - v_{13} + v_{14}$$
$$\frac{d}{dt}x_7 = v_{13} - v_{14} - v_{15} - v_{16} - v_{19} - v_{21} - v_{23}$$
$$\frac{d}{dt}x_8 = v_{16} - v_{17} - v_{18}$$
$$\frac{d}{dt}x_9 = v_{23} - v_{24} - s_3 + s_4$$
$$\frac{d}{dt}x_{10} = s_3 - s_4 - s_9$$
$$\frac{d}{dt}x_{11} = s_5 - s_6 - s_9 + s_{10}$$
$$\frac{d}{dt}x_{12} = s_9 - s_{10} - s_{11}$$
$$\frac{d}{dt}x_{13} = s_7 - s_8 - s_{11} + s_{12}$$
$$\frac{d}{dt}x_{14} = s_{11} - s_{12} - s_{15}$$
$$\frac{d}{dt}x_{15} = s_{13} - s_{14} - s_{15} + s_{16}$$
$$\frac{d}{dt}x_{16} = s_{15} - s_{16} - s_{17}$$
$$\frac{d}{dt}x_{17} = s_{17} - s_{18}$$
$$\frac{d}{dt}xu_1 = v_{19} - v_{20} - v_3{}' + v_4$$
$$\frac{d}{dt}xu_2 = v_{21} - v_{22}$$
$$\frac{d}{dt}xu_3 = -v_3 + v_4$$
$$\frac{d}{dt}xu_4 = -s_3 + s_4 - s_{19} \qquad (1)$$

Similarly, CAF dynamics can be described by mathematical equations in (2) as follows:

$$\frac{d}{dt}x_1 = v_1 - v_2 - v_3 - v_3{}' + v_4$$

$$\frac{d}{dt}x_2 = {v_3}' + v_3 - v_4 - v_9$$
$$\frac{d}{dt}x_3 = v_5 - v_6 - v_9 + v_{10} + v_{18}$$
$$\frac{d}{dt}x_4 = v_7 - v_8 - v_{11} + v_{12}$$
$$\frac{d}{dt}x_5 = v_9 - v_{10} - v_{11} + v_{12} - v_{18} - z_1 + z_2$$
$$\frac{d}{dt}x_6 = v_{11} - v_{12} - v_{13} + v_{14}$$
$$\frac{d}{dt}x_7 = v_{13} - v_{14} - v_{15} - v_{16} - v_{19} - v_{21} - v_{23}$$
$$\frac{d}{dt}x_8 = v_{16} - v_{17} - v_{18} + w_{20}$$
$$\frac{d}{dt}x_9 = v_{21} - v_{22} - {w_3}' + w_4$$
$$\frac{d}{dt}x_{10} = w_1 - w_2 - w_3 - {w_3}' + w_4$$
$$\frac{d}{dt}x_{11} = {w_3}' + w_3 - w_4 - w_7$$
$$\frac{d}{dt}x_{12} = w_5 - w_6 - w_7 + w_8$$
$$\frac{d}{dt}x_{13} = w_7 - w_8 - w_{11} + w_{12}$$
$$\frac{d}{dt}x_{14} = w_9 - w_{10} - w_{11} + w_{12}$$
$$\frac{d}{dt}x_{15} = w_{11} - w_{12} - w_{16} + w_{17} - w_{21} - w_{24} - z_1 + z_2$$
$$\frac{d}{dt}x_{16} = w_{16} - w_{17} - w_{18} - w_{20} - w_{13} + w_{14}$$
$$\frac{d}{dt}x_{17} = w_{13} - w_{14} - w_{15}$$
$$\frac{d}{dt}x_{18} = w_{18} - w_{19} - w_{21}$$
$$\frac{d}{dt}x_{19} = w_{22} - w_{23} - w_{24} - w_{15}$$
$$\frac{d}{dt}x_{20} = z_1 - z_2 - z_3 + z_4$$
$$\frac{d}{dt}x_{21} = z_3 - z_4 - z_5$$
$$\frac{d}{dt}x_{22} = z_5 - z_6$$
$$\frac{d}{dt}xu_1 = -v_3 + v_4$$
$$\frac{d}{dt}xu_2 = -w_3 + w_4$$
$$\frac{d}{dt}xu_3 = v_{19} - v_{20} - {v_3}' + v_4$$
$$\frac{d}{dt}xu_4 = v_{23} - v_{24} \qquad (2)$$

Equation (3) shows the PDEs for signaling molecules. In this equations you can find complete form of $f_i$ dynamics for each signaling molecules.

$$\begin{cases} \frac{d}{dt} xu_1(t,\vec{r}) = D_1 \Delta xu_1(t,\vec{r}) - \left(0.0051 + 0.5x_{23}^2(t)\right) xu_1(t,\vec{r}) + 0.24\, x_{24}(t) + 0.12 x_{29}(t) \\ \frac{d}{dt} xu_2(t,\vec{r}) = D_2 \Delta xu_2(t,\vec{r}) - 0.029 xu_2(t,\vec{r}) + 0.091 x_{29}(t) \qquad in \quad \Omega \times (0,T] \\ \frac{d}{dt} xu_3(t,\vec{r}) = D_3 \Delta xu_3(t,\vec{r}) - \left(0.0051 + 0.5x_1^2(t)\right) xu_3(t,\vec{r}) + 0.24\, x_2(t) + 0.12\, x_7(t) \\ \frac{d}{dt} xu_4(t,\vec{r}) = D_4 \Delta xu_4(t,\vec{r}) - 0.041 xu_4(t,\vec{r}) + 0.861\, x_7(t) \end{cases} \tag{3}$$

The initial values of all the signaling molecules are the same and is equal to 0.1 nMol.

Table S5: Diffusion coefficients of signaling molecules in the model

| **Diffusion Coefficient** | **Value** | **Reference** |
|---|---|---|
| $D_{TGF\beta}$ | $6 \times 10^{-5}\ cm^2/min$ | [62] |
| $D_{LIF}$ | $1.62 \times 10^{-5}\ cm^2/min$ | [93] |
| $D_{CXCL12}$ | $1.044 \times 10^{-4}\ cm^2/min$ | [94] |
| $D_{SNAIL}$ | $9 \times 10^{-7}$ cm $^2$ /min | [95] |

Table S6: Goodness of fit criteria for performance evaluation of the dynamic model of cancer cell

| **Molecule** | **Mean Error** | **NRMSE** | **$R^2$** |
|---|---|---|---|
| SMAD7 | 0.0085 | 0.0629 | 0.91 |
| TGFβ | -0.0012 | 0.0025 | 0.99 |
| LIF | 0.0056 | 0.0136 | 0.92 |
| CXCL12 | 0.0045 | 0.0025 | 0.99 |

Table S7: Goodness of fit criteria for performance evaluation of the dynamic model of CAF

| **Molecule** | **Mean Error** | **NRMSE** | **$R^2$** |
|---|---|---|---|
| SMAD7 | -0.0325 | 0.0629 | 0.67 |
| TGFβ | 0.0013 | 0.0017 | 0.99 |
| LIF | 0.0004 | 0.0602 | 0.87 |
| CXCL12 | 0.02 | 0.0215 | 0.86 |

Table S8: Goodness of fit criteria for performance evaluation of the biomechanical model of cancer cells movement

| **Statistic feature** | **Value** |
|---|---|
| $R^2$ | 0.991 |
| Mean Error | 0.0063 |
| NRMSE | 0.0151 |

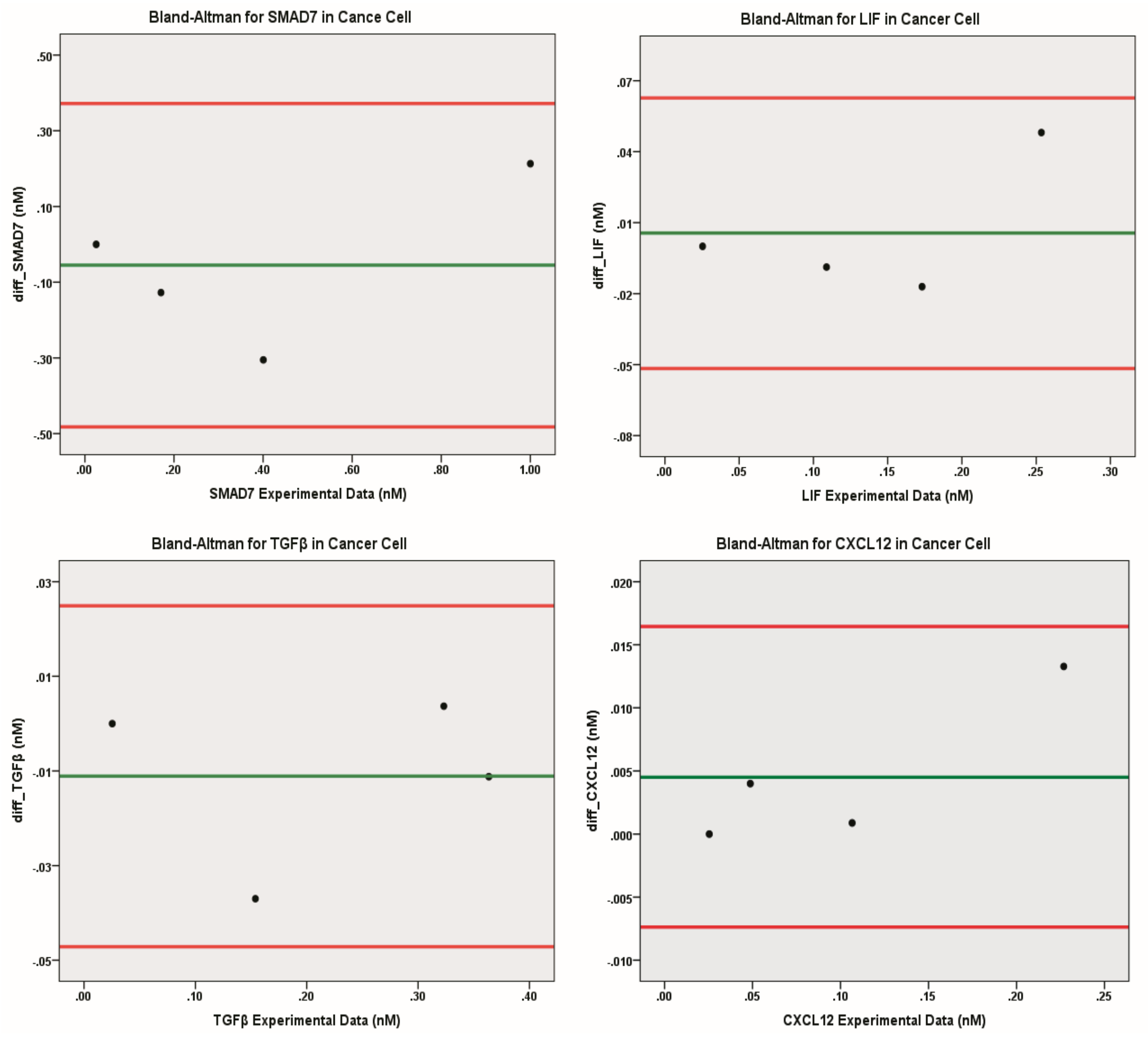


Fig S1: Bland-Altman graph for outputs of the dynamical model of cancer cell. The black dots show the value of difference between experimental data and simulation output of the model. The green line shows the mean value for the mentioned difference and the red lines indicate the $mean \pm 1.96sd$ in which sd is the standard deviation of the difference value between experimental data and simulation results.

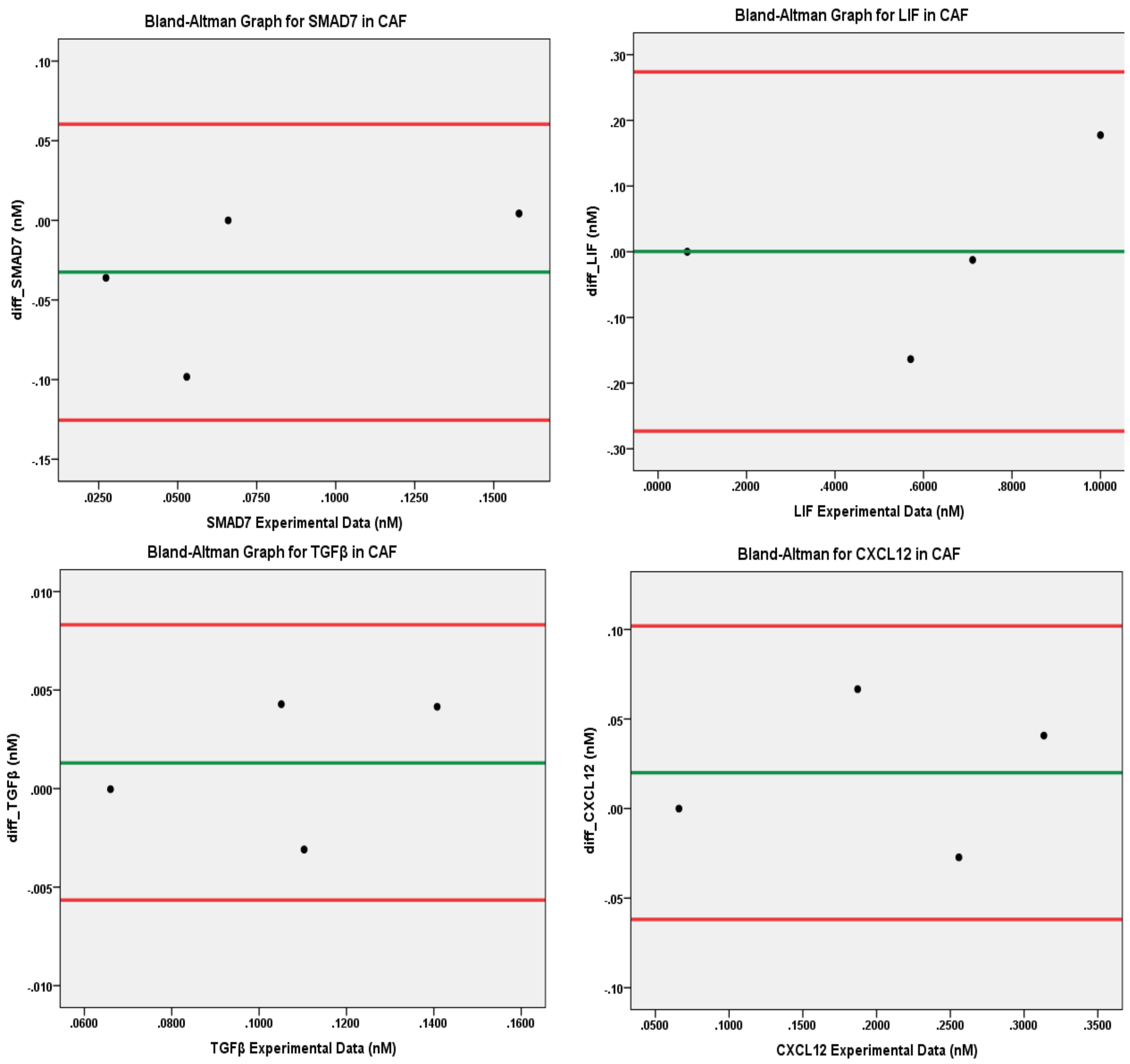


Fig S2: Bland-Altman graph for outputs of the dynamical model of CAF. The black dots show the value of difference between experimental data and simulation output of the model. The green line shows the mean value for the mentioned difference and the red lines indicate the $mean \pm 1.96sd$ in which sd is the standard deviation of the difference value between experimental data and simulation results.

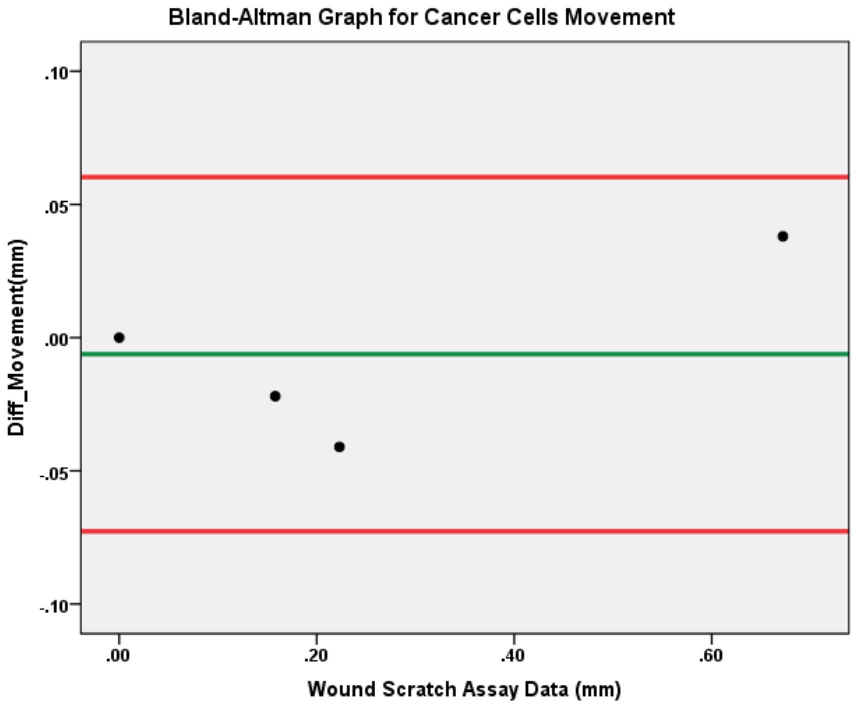


Fig S3: Bland-Altman graph for the biomechanical model of cancer cells movement. The black dots show the value of difference between experimental data and simulation output of the model. The green line shows the mean value for the mentioned difference and the red lines indicate the $mean \pm 1.96sd$ in which sd is the standard deviation of the difference value between experimental data and simulation results.

Table S9: Constants and parameters in forces shown in Table 3.

| **Parameter** | **Definition** | **Value** |
|---|---|---|
| $R$ | Cancer cell Raidus | 8 µm [66] |
| $E_i$ | Young's moduli | 1 kPa [18] |
| $\nu_i$ | Poisson number | $\frac{1}{3}$ [18] |
| $W$ | Adhesion energy | 200 µN/m [18] |
| $\chi$ | Haptotaxis coefficient | 0.018 [62] |
| $\eta$ | Strength of active force | 6000 µN [70] |
| $n$ | Hill coefficient | 3 [70] |
| $\Gamma^{cs}$ | Friction coefficient of cancer cell and TME | 24 $\mu Nmin/cm^{-1}$ [18] |

| | | |
|---|---|---|
| $r_i$ | Position vector of cell i | - |
| $d_{ij} = \lVert r_i - r_j \rVert$ | Distance of cells i and j | - |
| $\delta_{ij} = R_i + R_j - d_{ij}$ | Overlap between cells i and j | - |
| $\hat{E} = (\frac{1-\nu_i^2}{E_i} + \frac{1-\nu_j^2}{E_j})^{-1}$ | Defined constant in repulsive force between cells i and j | - |
| $\hat{R} = (\frac{1}{R_i} + \frac{1}{R_j})^{-1}$ | Defined constant in repulsive force between cells i and j | - |
| $A$ | Concentration of effective metastasis factor in active force | - |
| $A_0$ | Initialization threshold of effective factor in active force | - |
| $V$ | Cancer cell velocity | - |

## Sensitivity Analysis:

Table S10: Sensitivity analysis results for selected parameters of the dynamic models of cancer cell and CAF. The parameters are sorted based on mean values.

| Parameter | Mean value | Variance Value |
|---|---|---|
| ${k_9}^+$ | 0.2063 | 1.2e-3 |
| ${k_1}^+$ | 0.1557 | 2.1e-3 |
| ${k_8}^+$ | 0.1413 | 1.9e-3 |
| ${k_1}^-$ | 0.1188 | 6.31e-2 |
| ${k_{8i}}^-$ | 0.1181 | 1.8e-3 |
| ${p_{10}}^-$ | 0.1063 | 1.7e-3 |
| ${k_{10}}^+$ | 0.0835 | 4.75e-5 |
| ${k_{11}}^+$ | 0.0631 | 4.34e-4 |
| ${k_8}^-$ | 0.0580 | 1.01e-4 |
| ${k_9}^-$ | 0.0533 | 6.45e-5 |
| ${h_2}^+$ | 0.0484 | 6.97e-3 |
| ${k_2}^+$ | 0.0465 | 3.27e-4 |
| ${k_{10}}^-$ | 0.0423 | 5.15e-3 |
| ${k_2}^-$ | 0.0303 | 1.31e-5 |
| ${p_2}^-$ | 0.0302 | 3.28e-5 |
| ${h_2}^-$ | 0.0297 | 1.1e-3 |
| ${k_{11}}^-$ | 0.0192 | 2.15e-3 |
| ${p_2}^+$ | 0.0170 | 2.38e-6 |
| ${h_{10}}^+$ | 0.0029 | 1e-3 |

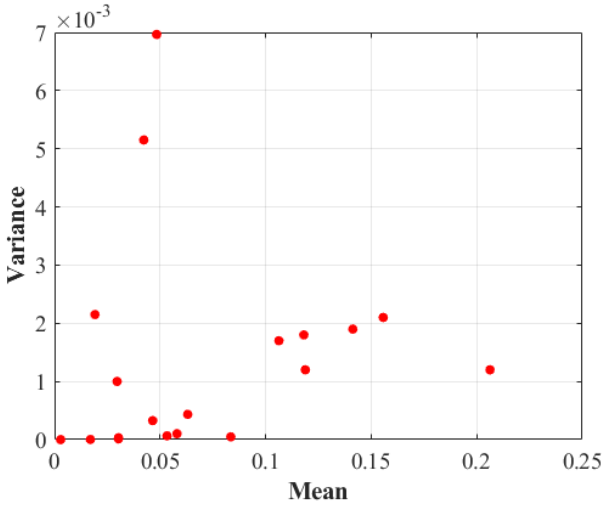


Fig S4: The Morris sensitivity analysis result of the dynamic model of cancer cells and CAF. The axes indicate the mean and variances of the element effects for each parameter.
